# Complete Genome Sequencing and Comparative Analysis of the Clinically-Derived *Apiotrichum mycotoxinivorans* Strain GMU1709

**DOI:** 10.3389/fcimb.2022.834015

**Published:** 2022-02-04

**Authors:** Liang Peng, Chen-Fei Liu, Hong Wu, Hai Jin, Xiao-Yan Deng, Li-Ting Zeng, Yi Xiao, Cong Deng, Zhi-Kai Yang

**Affiliations:** ^1^ The Fifth Affiliated Hospital of Guangzhou Medical University, Guangzhou, China; ^2^ KingMed School of Laboratory Medicine, Guangzhou Medical University, Guangzhou, China; ^3^ The Second Affiliated Hospital of Guangzhou Medical University, Guangzhou, China

**Keywords:** *Apiotrichum mycotoxinivorans*, complete genome sequencing, whole genome-based phylogeny, comparative genome analysis, pathogenicity, drug resistance

## Abstract

Over the past decade, *Apiotrichum mycotoxinivorans* has been recognized globally as a source of opportunistic infections. It is a yeast-like fungus, and its association as an uncommon pulmonary pathogen with cystic fibrosis patients has been previously reported. Immunocompromised patients are at the highest risk of *A. mycotoxinivorans* infections. Therefore, to investigate the genetic basis for the pathogenicity of *A. mycotoxinivorans*, we performed whole-genome sequencing and comparative genomic analysis of *A. mycotoxinivorans* GMU1709 that was isolated from sputum specimens of a pneumonia patient receiving cardiac repair surgery. The assembly of Oxford Nanopore reads from the GMU1709 strain and its subsequent correction using Illumina paired-end reads yielded a high-quality complete genome with a genome size of 30.5 Mb in length, which comprised six chromosomes and one mitochondrion. Subsequently, 8,066 protein-coding genes were predicted based on multiple pieces of evidence, including transcriptomes. Phylogenomic analysis indicated that *A. mycotoxinivorans* exhibited the closest evolutionary affinity to *A. veenhuisii*, and both the *A. mycotoxinivorans* strains and the formerly *Trichosporon cutaneum* ACCC 20271 strain occupied the same phylogenetic position. Further comparative analysis supported that the ACCC 20271 strain belonged to *A. mycotoxinivorans*. Comparisons of three *A. mycotoxinivorans* strains indicated that the differences between clinical and non-clinical strains in pathogenicity and drug resistance may be little or none. Based on the comparisons with strains of other species in the *Trichosporonaceae* family, we identified potential key genetic factors associated with *A. mycotoxinivorans* infection or pathogenicity. In addition, we also deduced that *A. mycotoxinivorans* had great potential to inactivate some antibiotics (*e.g.*, tetracycline), which may affect the efficacy of these drugs in co-infection. In general, our analyses provide a better understanding of the classification and phylogeny of the *Trichosporonaceae* family, uncover the underlying genetic basis of *A. mycotoxinivorans* infections and associated drug resistance, and provide clues into potential targets for further research and the therapeutic intervention of infections.

## Introduction

*Apiotrichum mycotoxinivorans* (formerly *Trichosporon mycotoxinivorans*) is a basidiomycete yeast of the *Trichosporonaceae* family (Peng et al., 2019). It was first isolated from the hindgut contents of Australian lower termites (Schatzmayr et al., 2003), then identified as a new species of the genus *Trichosporon* based on the phylogenetic analysis of 26S rDNA, and named *T. mycotoxinivorans* for its beneficial detoxifying properties on the mycotoxins zearalenone and ochratoxin A (Molnar et al., 2004). Owing to the limitations of phenotype/26S rDNA-based classifications, Liu et al. (2015) proposed an updated classification for *Tremellomycetes* based on a seven-gene phylogeny in which *T. mycotoxinivorans* was assigned to the genus *Apiotrichum* under the name *A. mycotoxinivorans*. With the advances in sequencing technology, an increasing number of genomes belonging to the *Trichosporonaceae* family have been sequenced (Wang et al., 2016; Close and Ojumu, 2016; Gil et al., 2018; Gorte et al., 2019; Sun et al., 2020) and released recently by different laboratories worldwide that provides an unprecedented opportunity to identify some potential controversial phylogenetic relationships in current classifications extensively (Rodríguez et al., 2017).

Although *A. mycotoxinivorans* is universally regarded as a validated anti-mycotoxin feed additive (Khalel et al., 2012), it has been gradually recognized as an opportunistic pathogen with a known propensity for patients with cystic fibrosis. As early as 2009, Hickey et al. reported the first case of human disease caused by *A. mycotoxinivorans* in a non-transplant patient with cystic fibrosis. Later, Hirschi et al. (2012) reported the first case of disseminated fungal co-infection caused by *A. mycotoxinivorans* and another fungus, which emerged after liver and lung transplantation in a patient with cystic fibrosis. Subsequently, a case series of chronic *A. mycotoxinivorans* infection has been reported in patients with cystic fibrosis (Shah et al., 2014; Goldenberger et al., 2016). Unlike previous studies, Dabas et al. (2017) reported the emergence of *A. mycotoxinivorans* in three cases of bloodstream infections in a retrospective study. Almeida et al. (2017) detected *A. mycotoxinivorans* from peritoneal fluid in the early stage of disseminated infection and further from the blood in the late stage of the infection. Marcelo and Farooq (2018) presented the first case of *A. mycotoxinivorans* dissemination in the brain of a patient who had positive blood and stool cultures for *A. mycotoxinivorans*. In 2019, we also isolated and identified the *A. mycotoxinivorans* GMU1709 strain from sputum specimens of a pediatric patient with pneumonia, and then reported its morphological, biochemical, and molecular characteristics (Peng et al., 2019). Sadamatsu et al. (2020) reported a rare case of pulmonary co-infection with *A. mycotoxinivorans* and *Cryptococcus neoformans*. Although the aforementioned studies have adequately described the clinical cases of human disease caused by this opportunistic fungal pathogen, including the antifungal susceptibility patterns, different infection modes, and clinical consequences, the molecular genetic bases closely related to its infection, pathogenicity, and drug resistance remain largely unexplored.

As is well known, genome sequencing refers to adopting sequencing technology to obtain the whole-genome sequence of an organism, which is an important step toward associating genotypes with phenotypic characters (Verma et al., 2017). By investigating the genome database of the National Center for Biotechnology Information (NCBI), we deduced that more than 30 genomes of *Trichosporonaceae* have been recently published. Before performing this study, no genome sequence of *A. mycotoxinivorans* has been sequenced or released, although this yeast has multiple important biological properties, especially pathogenicity. Its evolutionary relationships with other species in *Trichosporonaceae* also need to be assessed at higher phylogenetic resolutions. Hence, we sequenced the genome and transcriptome of the clinically-derived *A. mycotoxinivorans* strain (GMU1709), and obtained and published its complete genome sequence for the first time on March 15, 2020. Soon after, from the NCBI genome database, we retrieved the genome of environmentally derived *A. mycotoxinivorans* strain (CICC 1454) that was published on May 27, 2020, which further provided important support for the intraspecific comparative analysis. We found that Sun et al. (2020) mainly determined the possible zearalenone-degradation enzymes by their bioinformatic method after they assembled and annotated the genome of CICC 1454 strain. Therefore, this study attempted to uncover the pathogenic genetic basis of *A. mycotoxinivorans* and the genetic differences between clinical and non-clinical strains as well as to provide a clearer phylogenetic relationship with other species. The results of this study would provide insights into the prevention and control of *A. mycotoxinivorans* infection and how to avoid the influence of its pathogenicity on its application in detoxification.

## Materials and Methods

### DNA/RNA Extraction, Sequencing, and Assembly

*A. mycotoxinivorans* GMU1709 (Peng et al., 2019) was cultured at 37°C in a malt extract medium for 48 h. Cells were collected by centrifugation at 4500 ×g for 10 min and broken by grinding under liquid nitrogen for 20 min. Genomic DNA was extracted according to the cetyltrimethylammonium bromide protocol (Kim et al., 2010). Next, three different libraries were constructed by the Biomarker Technologies, Inc., according to the manufacturer’s standard procedures, in which the paired-end library with a 350-bp insert size was sequenced on an Illumina HiSeq platform, the 30-kb library was sequenced using a GridION DNA sequencer, and the Hi-C library was sequenced with a 150-bp read length (PE 150) on the Illumina platform. Quality control was performed on the sequencing data as follows: For Illumina reads, adaptors and low-quality bases located at both read ends were first trimmed; then, the corresponding paired-end reads were abandoned if any read contained more than 5% unknown bases or was shorter than 30 bp after quality-based trimming; PCR duplicates were routinely discarded using SAMTools (Li et al., 2009); For Nanopore reads, reads with mean quality < 7 or length < 500 bp were discarded; For Hi-C reads, the HiC-Pro software (Servant et al., 2015) was employed with default settings to perform quality control filtering. GCE 1.0.0 (ftp://ftp.genomics.org.cn/pub/gce) was adopted to estimate the genome size. Error correction of Nanopore reads was conducted using Canu v1.5 (Koren et al., 2017), and the corrected reads were assembled into contigs using WTDBG2 with defaults (Ruan et al., 2020). Illumina paired-end reads were mapped to the assembly, based on which dubious bases were corrected by the Pilon pipeline (Walker et al., 2014) to achieve a high-quality genome. Genomic completeness was estimated using the BUSCO value (Simão et al., 2015) and the ratio of Illumina reads mapped back to the genome. Finally, LACHESIS was used to determine the order and orientation of contigs by mapping the Hi-C reads to contigs (Burton et al., 2013).

Total RNA was extracted from GMU1709 using a TRIzol reagent (Invitrogen, Carlsbad, CA, USA) according to the manufacturer’s instructions. cDNA was synthesized from RNA using the PrimerScript RT reagent kit (Takara, Dalian, Liaoning, China). The cDNA library was prepared using the Illumina TreSeq RNA Sample Preparation Kit (Illumina, Inc., San Diego, CA, USA) following the manufacturer’s protocol, in which oligo-dT beads were used to capture mRNA, the first strand DNA was synthesized using oligo dT primers. RNA-seq was conducted on an Illumina HiSeq 2500 platform with a 150-bp paired-end strategy. Transcriptome reads were mapped to the genome sequences using Hisat2 v2.0.4 (Pertea et al., 2016). Transcripts were assembled using Stringtie v1.2.3 (Pertea et al., 2016), and then adopted to provide evidence for subsequent *ab initio* gene prediction.

### Gene Prediction and Functional Annotation

Before gene prediction, repetitive DNA sequences were identified by using RepeatModeler open-1.0.11 (Smit et al., 2015) and RepeatMasker v4.0.6 (Tarailo-Graovac and Chen, 2009) with default and masked. Gene prediction was performed as follows: First, we separately employed Genscan (Burge and Karlin, 1997), Augustus v2.4 (Stanke and Waack, 2003), GeneID v1.4 (Blanco et al., 2007), GlimmerHMM v3.0.4 (Majoros et al., 2004), and SNAP v2006-07-28 (Korf, 2004) to perform the *ab initio* gene prediction. Next, GeMoMa v1.3.1 (Keilwagen et al., 2016) was adopted for homology-based gene prediction, in which the protein sets from *A. porosum*, *Cutaneotrichosporon oleaginosum*, and *T. asahii* were taken as references. Transcriptome-based gene prediction was performed using the TransDecoder v2.0 (Haas et al., 2013) and PASA v2.0.2 (Haas et al., 2008), respectively. Finally, EVM v1.1.1 (Haas et al., 2008) was used to improve the results derived from the aforementioned three methods, as well as integrate them into the final gene set. Regarding noncoding RNAs, tRNA genes were predicted by tRNAscan-SE (Lowe and Eddy, 1997), whereas rRNA genes and other ncRNA genes were predicted based on the Rfam database (Nawrocki et al., 2014) using Infernal v1.1 (Nawrocki and Eddy, 2013). The GenBlastA (She et al., 2009) was adopted to align the predicted proteins against expertly curated proteins from the Swiss-Prot database, and then the local version of the Genewise program (Birney et al., 2004) was utilized to identify the premature termination codon and frameshift mutation of homologous genes to determine these potential pseudogenes. The functions of the predicted genes were annotated by BLAST (Altschul et al., 1997, E-value cutoff of 1e-5) based on the NCBI non-redundant database (NR) (Deng et al., 2006). SignalP v4.0 (Petersen et al., 2011) was used to identify proteins with signal peptides, and the Transmembrane Helices Hidden Markov Model program (Krogh et al., 2001) was adopted to predict proteins with transmembrane helices. Proteins with signal peptides, but without transmembrane helices, were considered as potential secretory proteins.

### Whole Genome-Based Phylogenetic Analysis

We obtained all genome sequences of *Trichosporonaceae* (**Table 1**) from the NCBI GenBank database. Two *Takashimella* strains (*T. koratensis* JCM 12878 and *T. tepidaria* JCM 11965) in the family *Tetragoniomycetaceae*, which are closely related to the family *Trichosporonaceae*, were served as outgroups. Whole genome-based phylogenetic tree was constructed *via* the maximum-likelihood (ML) method as follows: First, Mugsy v1.2.3 (Angiuoli and Salzberg 2010) was adopted to identify the homologous regions, whereas the homologous blocks present in all genomes were extracted using a custom PERL script and then realigned using muscle v3.8.31 (Edgar 2004) with the default parameters. Next, non-gaped sites were concatenated into a new multiple sequence alignment using a custom PERL script. Subsequently, a phylogenetic tree was constructed using raxmlHPC-PTHREADS-AVX v8.2.10 (Stamatakis 2014) with the GTRGAMMA model of evolution (100 bootstrap replicates), including a subsequent search for the best-scoring ML topology. Finally, we employed the Interactive Tree of Life v4 (Letunic and Bork, 2019) to display this tree and its relevant information.

**Table 1:** Thirty-six strains from four genera and 25 species of the *Trichosporonaceae* family.

Organism name	Strain	Bioproject	Assembly	GenomeSize (bp)	GC Content	Sequence Number	Gap Number	Protein Number
*A. brassicae*	JCM 1599	PRJDB3695	GCA_001600295.1	23647732	0.565	16	62055	7057
*A. domesticum*	JCM 9580	PRJDB3573	GCA_001599015.1	24510922	0.585	28	31175	7123
*A. gamsii*	JCM 9941	PRJDB3703	GCA_001600315.1	24609388	0.611	29	44067	7563
*A. gracile*	JCM 10018	PRJDB3704	GCA_001600335.1	24114851	0.592	17	17924	7542
*A. laibachii*	JCM 2947	PRJDB3730	GCA_001600735.1	30616633	0.596	26	160980	8597
*A. montevideense*	JCM 9937	PRJDB3572	GCA_001598995.1	24872216	0.582	61	77900	7495
*A. mycotoxinovorans*	CICC 1454	PRJNA633776	GCA_013177335.1	30749651	0.576	7	0	7991
*A. mycotoxinovorans*	GMU1709	PRJNA610126	GCA_011290525.1	30456694	0.576	7	0	8069
*A. porosum*	DSM 27194	PRJNA531017	GCA_003942205.1	25479456	0.592	32	0	8202
*A. porosum*	JCM 1458	PRJDB3693	GCA_001600255.1	25989348	0.590	37	26976	8341
*A. veenhuisii*	JCM 10691	PRJDB3717	GCA_001600595.1	31617680	0.596	35	81669	8168
*C. arboriformis*	JCM 14201	PRJDB5900	GCA_002335565.1	19894493	0.606	28	300	6428
*C. oleaginosum*	ATCC 20509	PRJNA327102	GCA_001712445.1	19908169	0.606	16	45931	7166
*C. oleaginosum*	ATCC 20508	PRJNA475739	GCA_008065305.1	19820908	0.607	8	0	7272
*C. oleaginosum*	IBC0246	PRJNA342699	GCA_001027345.1	19835558	0.607	180	4840	7170
*C. curvatum*	SBUG-Y 855	PRJNA281029	GCA_001028165.1	16443618	0.594	354	0	5521
*C. cutaneum*	B3	PRJNA310294	GCA_001636075.1	38696417	0.603	592	0	12464
*C. cutaneum*	JCM 1462	PRJDB3729	GCA_001600715.1	23155501	0.620	98	395801	8208
*C. cyanovorans*	JCM 31833	PRJDB5903	GCA_002335625.1	19941766	0.580	90	932	5991
*C. daszewskae*	JCM 11166	PRJDB5901	GCA_002335585.1	17225847	0.610	12	223	6935
*C. dermatis*	JCM 11170	PRJDB3725	GCA_003116895.1	23337637	0.600	37	19242	7776
*C. mucoides*	JCM 9939	PRJDB3710	GCA_003116955.1	40782531	0.601	83	63375	12744
*T. akiyoshidainum*	HP2023	PRJNA428315	GCA_002973495.1	30040186	0.606	1061	20	8764
*T. asahii*	CBS 2479	PRJNA296794	GCA_000293215.1	24540311	0.590	78	236477	7779
*T. asahii*	CBS 8904	PRJNA172216	GCA_000299215.2	25299608	0.589	194	251919	7787
*T. asahii*	JCM 2466	PRJDB3696	GCA_001972365.1	24687929	0.594	36	74412	7587
*T. asahii*	N5 275 008G1	PRJNA471744	GCA_004026345.1	23418624	0.596	1022	49	7600
*T. coremiiforme*	JCM 2938	PRJDB3697	GCA_001752605.1	42353277	0.596	190	86193	13267
*T. cutaneum*	ACCC 20271	PRJNA313001	GCA_001613755.1	30717177	0.571	21	273242	7891
*T. faecale*	JCM 2941	PRJDB3698	GCA_001752585.1	24653913	0.602	32	67450	7909
*T. inkin*	ATCC 18020	PRJNA312557	GCA_004023515.1	17232077	0.616	6682	1	7277
*T. inkin*	JCM 9195	PRJDB3701	GCA_001752625.1	20339538	0.627	18	34588	6750
*T. ovoides*	JCM 9940	PRJDB3702	GCA_001752645.1	40321892	0.602	115	45776	12469
*V. humicola*	CBS 4282	PRJNA475686	GCA_008065275.1	22632906	0.628	21	139	8472
*V. humicola*	JCM 1457	PRJDB3692	GCA_001600235.1	22653840	0.627	10	35628	8487
*V. humicola*	UJ1	PRJDB6593	GCA_002897395.1	22626796	0.628	39	21491	8418

### Orthologous Gene Families and Synteny Analysis

Currently, 25 species and 36 strains from four genera (**Table 1**) in the *Trichosporonaceae* family have been sequenced; however, most of the gene or protein sets are not available from public databases, which limits our comparative analysis. Here, we downloaded all these genomes from the NCBI genome database and carried out gene prediction using Funannotate v1.7.4 (https://funannotate.readthedocs.io/en/latest/index.html). Subsequently, gene clustering analysis was conducted using the OrthoMCL software (Li et al., 2003) with the following parameters: percentMatchCutoff = 80, inflation value = 1.5, percentIdentityCutoff = 80, and blastPvalueCutoff = 1e-5. The output was transformed into matrix data using a custom PERL script and then clustered using heatmap.2 in R (Zhao et al., 2014). JCVI v1.0.1 was adopted to construct chromosome-level synteny block plots with default parameters (Tang et al., 2015). For inter-species comparisons, two species with the most complete genomes (the *A. brassicae* JCM 1599, *A. gracile* JCM 10018, *T. inkin* JCM 9195, *T. faecale* JCM 2941, *C. oleaginosum* ATCC 20508, and *C. daszewskae* JCM 11166) were selected from each of three most closely related genera. For intraspecific comparisons, we used the *A. mycotoxinovorans* CICC 1454 and GMU1709 strains, as well as the previously reported *Trichosporon cutaneum* ACCC 20271 strain (Wang et al., 2016). Genomic comparison analyses would provide further evidence for their evolutionary relationships.

### Identification of Virulence Genes and Resistance Genes

To identify the virulence and resistance genes in *A. mycotoxinivorans* and compare the intraspecific differences in pathogenic genetic basis between clinical and non-clinical strains and the interspecific differences between *A. mycotoxinivorans* and other *Trichosporonaceae* species (**Table 1**), the protein sequences of all these strains were aligned using the BLASTP command of DIAMOND (v2.0.11.149) (Buchfink et al., 2015) with an E-value cutoff of 1e-5 against the mycology antifungal resistance database (MARDy) (Nash et al., 2018), pathogen-host interactions database (PHI-base) (Urban et al., 2020), and comprehensive antibiotic database (CARD) (Jia et al., 2017). Group differences were analyzed using the Wilcox test, and the results were visualized using the pheatmap R package.

### Bacterial Survival Assay

The yeast *A. mycotoxinivorans* strain GMU1709 was cultured in malt extract medium at 35°C and 200 rpm for 24 h. Then, the culture was collected by centrifugation for 2 min at 4500 ×g. The cell pellet was washed with 0.9% saline for 3 times, and the absorbance of suspension at 600 nm was adjusted to 2.0. The cultured *Escherichia coli* strain ATCC 25922 in Luria-Bertani (LB) medium was treated similarly to that of the GMU1709, but the absorbance of 600 nm was adjusted to 0.5. The experiment was divided into three groups: group A of live fungi mixed with bacteria, group B of inactivated fungi (sterilization at 121°C for 15 min) mixed with bacteria, group C of bacteria suspended in saline. The fungi and bacteria were mixed in a ratio of 2:1. All the three groups were added with 10 µg/mL of tetracycline and incubated at 35°C for 2 h, then the samples of three groups were collected respectively and cultured on LB agar plates with 10 times gradient dilution for survival bacterial counting. The relative survival rate of *E. coli* was expressed using the following equation: (survival bacteria of group B or C/survival bacteria of group A) ×100%. Experiments were conducted in triplicate.

## Results

### Assembly and Characterization of *A. mycotoxinivorans* GMU1709 Genome

**Figure 1:**
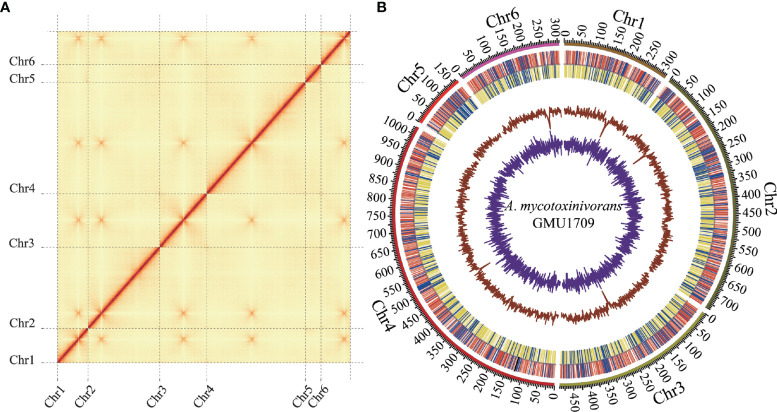
Hi-C assisted assembly of *A. mycotoxinivorans* GMU1709 genome using LACHESIS software. **(A)** Hi-C heatmap showing chromosomal interactions under a resolution of 20 kB. Color darkness represent the number of validly mapped Hi-C read pairs between any two bins (20 kB). The contact between centromeres in chromosomes results in strong enrichment of centromere-to-centromere Hi-C links. **(B)** Circos plot illustrating genomic characteristics. Tracks from outside to the inner correspond to the genome (10 kB), gene distributions on the forward (red) and reverse (yellow) strands (tRNA and rRNA genes marked in blue and black, respectively), GC ratio, and GC skew.

In this study, we completed the sequencing, *de novo* assembly, and annotation of clinically-derived *A. mycotoxinivorans* GMU1709 genome. First, approximately 11.6 Gb of high-quality genomic data was generated using the Illumina platform. Based on these data, the genome size was estimated to be 32.7 Mb (actual) or 29.2 (fitted) Mb *via* a 21-mer analysis (**Figure S1**). Next, 8.1 Gb of long-read data (~231X depth) with an average length of 29.8 kB was produced by the Nanopore platform (**Table S1** and **Figure S2**). *Via* the *de novo* genome assembly, we obtained seven ungapped contigs with a total length of 30.5 Mb (**Table 2**), an average GC content of 57.6%, and an N50 length of approximately 7.4 Mb. Subsequently, Illumina reads were used to correct the assembly. Based on Hi-C reads (10.6 Gb), six out of seven contigs (accounting for 99.9% of the total bases) were anchored to six chromosomes. The only one unallocated was found to be the mitochondrial sequence (16 895 bp) by BLAST against the NT database. It was reported that the combination of Illumina and Nanopore sequencing reads allowed for producing the telomere-to-telomere chromosome assemblies (Bliznina et al., 2021). However, we didn’t detect typical telomere sequences from the GMU1709 genome according to previously reported rules, *e.g*. (CCCTAA/TxAyGz)n (Peska and Garcia, 2020; Rahnama et al., 2021). Meanwhile, we also failed to find telomere sequences from the CICC 1454 genome. Current evidence has suggested that the interchromosomal contacts would result in strong enrichment of centromere-to-centromere Hi-C links (Duan et al., 2012). Using the Hi-C heatmap (**Figure 1A**), five out of six chromosomes were determined to contain clear centromeric signatures. However, according to Varoquaux et al. (2015), the remaining one may be influenced by the sequence around centromere, thus showing indiscernible centromeric signature. We also examined the mapping ratio of Illumina reads against the genome and determined that 92.2% of the reads were properly mapped (**Table S2**). BUSCO analysis indicated that 95.2% of the expected fungal genes were present in the gene set of GMU1709. These results suggest a high-quality chromosomal-level genome assembly (**Figure 1B**). Using the RepeatModeler and RepeatMasker prediction, we totally identified 1 416 118 bp of repetitive sequences. Specifically, there were 340 424 bp, 1793 bp, 1612 bp, 937 936 bp, and 134 423 bp of interspersed repeats (including the short interspersed nuclear elements (SINEs), long interspersed nuclear elements (LINEs), long terminal repeat (LTR) retrotransposons, DNA elements, and others), small RNA, satellites, simple repeats, and low complexity repeats, respectively. Compared to most of other strains, we found a high proportion of simple repeats and unclassified interspersed repeats in the genomes of *A. mycotoxinivorans* strains (**Figure S3**). Using homology-based, RNA-seq-based, and *ab initio* methods, we identified 8066 protein-coding genes. The average length of protein-coding genes was 2371.5 bp, and they averagely contained 4.8 exons (average length: 416.6 bp) and 3.8 introns (average length: 99.4 bp). In this genome, there were two rRNAs, 917 tRNA, 17 ncRNAs, and eight pseudogenes. A total of 89.6% of proteins were annotated, and 5.6% of them were identified as putative secretory proteins.

**Table 2:** Statistics for the contig length of the *A. mycotoxinivorans* GMU1709 genome assembly.

Sequence Name	Sequence Length (bp)
contig1	3,171,597
contig2	7,427,248
contig3	4,906,019
contig4	10,238,178
contig5	1,646,260
contig6	3,050,497
contig7	16,895

### Phylogenetic Relationship

**Figure 2:**
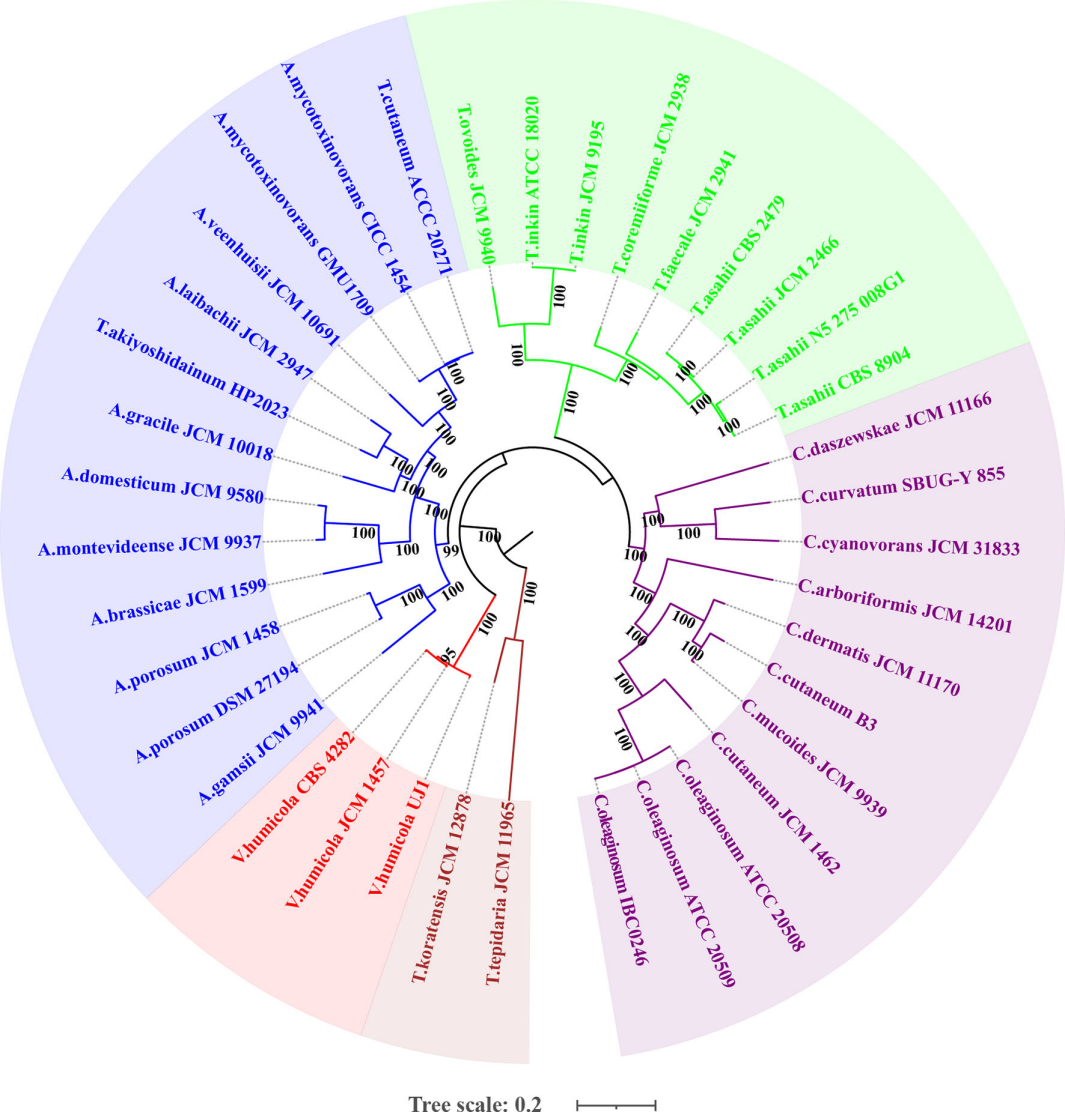
Maximum likelihood (ML)-based phylogenomic analysis of thirty-six *Trichosporonaceae* members. The ML tree is inferred from the concatenated non-gaped sites of multiple whole-genome alignments. The phylogeny is generated using raxmlHPC-PTHREADS-AVX version 8.2.10 based on the GTRGAMMA model and 100 bootstrap replicates. The genera *Apiotrichum*, *Trichosporon*, *Cutaneotrichosporon*, *Vanrija* in the family *Trichosporonaceae* and the genus *Takashimella* (outgroup) in *Tetragoniomycetaceae* are highlighted in blue, green, purple, red, and brown respectively.

The whole genome-based phylogenetic tree (**Figure 2**) demonstrated that the formerly called *T. mycotoxinivorans* undoubtedly belongs to the genus *Apiotrichum*, which is consistent with the findings of Liu et al. (2015). *T. cutaneum* ACCC 20271, *A. mycotoxinivorans* GMU1709, and CICC 1454 strains were located almost in the same phylogenetic position (**Figure 2** **)**, thus indicating that the ACCC 20271 strain belongs to the *A. mycotoxinivorans* species. Aliyu et al. (2020) classified the ACCC 20271 strain into the genus *Apiotrichum*, but did not subdivide it into *A. mycotoxinivorans* because the genome of *A. mycotoxinivorans* is yet to be published. In addition, the *T. akiyoshidainum* HP2023 strain should be reclassified as *A. akiyoshidainum*, which is consistent with the findings of Aliyu et al. (2020), while C. Cutaneum B3 and JCM 1462 strains are located in different branches of the genus Cutanetrichosporon, indicating that they belong to different species. Hence, our analysis presented highly reliable evolutionary relationships among different *Trichosporonaceae* species, together with the discovery of previously misclassified strains.

### Gene Clustering Analysis

**Figure 3:**
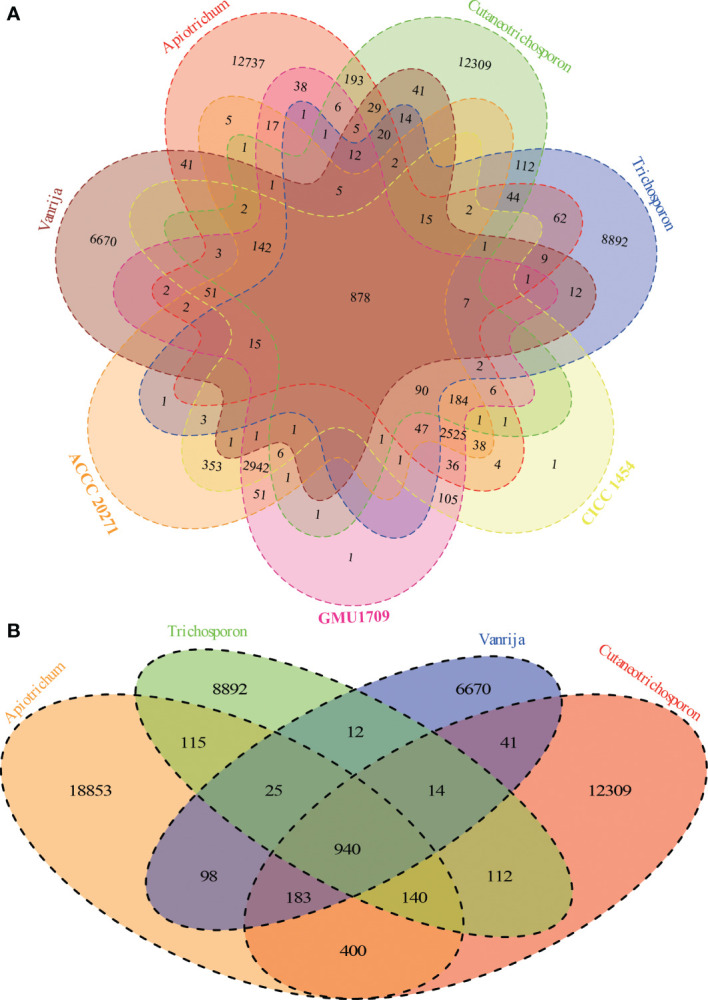
Relationships among *A. mycotoxinivorans* strains and different *Trichosporonaceae* genera based on 48804 orthologous groups. **(A)** Venn diagram presents the relationships between three *A. mycotoxinivorans* strains and four *Trichosporonaceae* genera. *A. mycotoxinivorans* is not included in *Apiotrichum*. **(B)** Venn diagram presents the relationships between four *Trichosporonaceae* genera.

Via the Markov clustering analysis of protein sets (294 280 proteins in total) of 36 strains in four genera of *Trichosporonaceae* (**Table 1**), 76% (224 590) of the proteins were clustered into 48 804 orthologous groups, of which 2 (corresponding to the fibrillarin and DEAD/DEAH box helicase, respectively) and 395 were present in all strains as single and multiple copies, respectively. Based on the hierarchical cluster analysis of gene presence and absence patterns across 36 strains, we identified that different strains of the same species had nearly identical patterns of gene presence and absence (**Figure S4**), which further supported that all three strains (GMU1709, CICC 1454, and ACCC 20271) belong to the same species. According to the Venn relationships of the aforementioned three strains and four *Trichosporonaceae* genera (*Apiotrichum*, *Cutaneotrichosporon*, *Trichosporon*, and *Vanrija*), we found that only 878 orthologous groups were shared by them; 2942 groups were exclusively shared by these three strains, whereas few groups were shared by these three strains and any of the other genera, except the *Apiotrichum* genus (**Figure 3A**). This also supported the position that the formerly named *T. mycotoxinivorans* belongs to the *Apiotrichum* genus. In addition, we also determined that the *Apiotrichum* genus possessed the largest number of groups and genus-specific groups, followed by the *Cutaneotrichosporon* and *Trichosporon* genera, and the least abundant *Vanrija genus* (**Figure 3B**), which may be influenced by the number of strains or species in each genus (**Table 1**). In summary, these results strongly support the whole genome-based phylogenetic relationship.

### Syntenic Relationship

**Figure 4:**
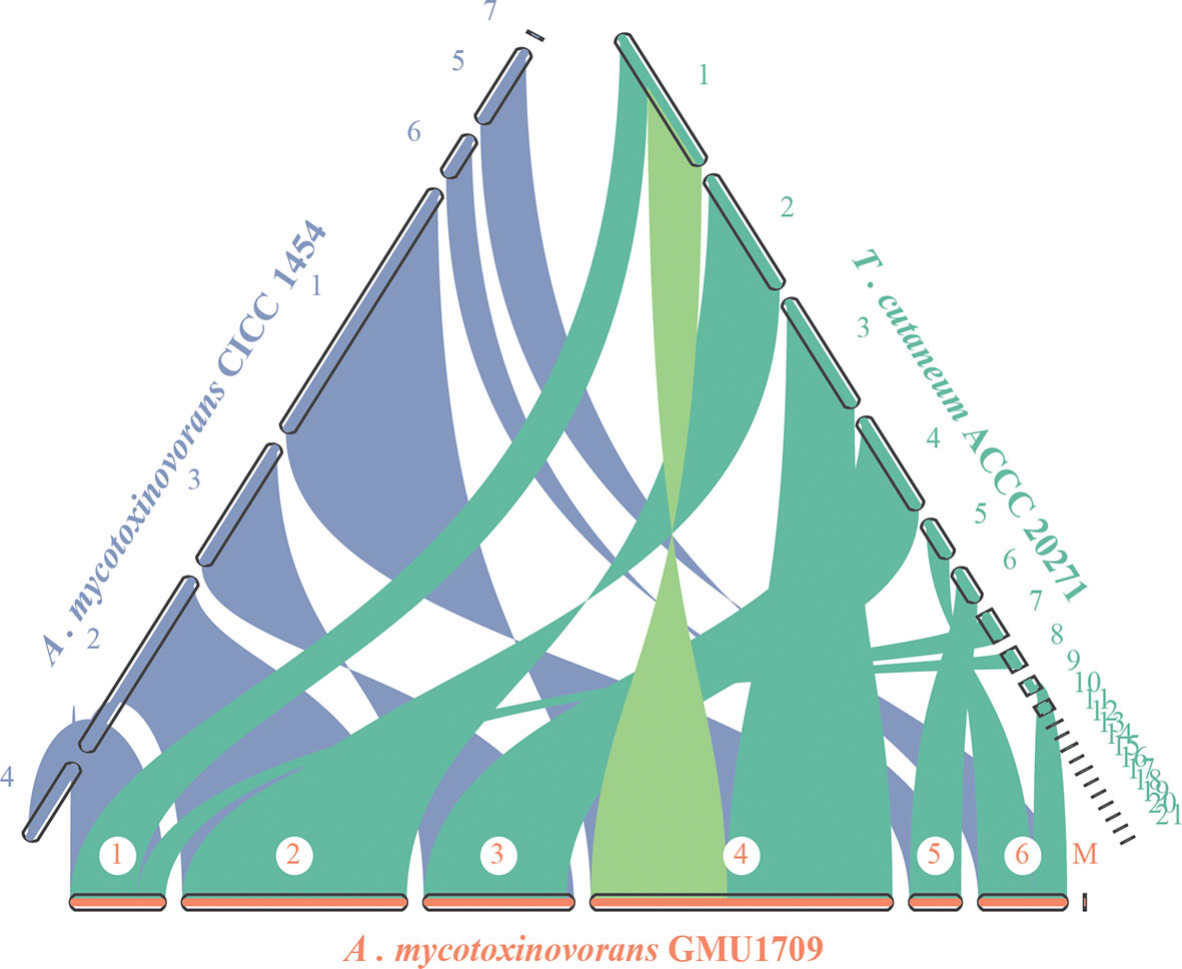
Synteny analysis of *A. mycotoxinivorans* GMU1709 and CICC 1454 and formerly *T. cutaneum* ACCC 20271 using JCVI v1.0.1 software. Synteny patterns show that each genomic block of GMU1709 strain aligns with one genomic block of CICC 1454 and ACCC 20271 strains. Dubious region in one scaffold of ACCC 20271 genome assembly is highlighted in light green.

The evolutionary relationship of chromosomes between strains was determined using the genomic synteny block analysis. Our results indicated strong genomic collinearity among GMU1709, CICC 1454, and ACCC 20271 strains (**Figures S5A–C**), except for an inconsistent event (highlighted in **Figure 4**) in one genomic sequence of the ACCC 20271 strain. In fact, the genome of ACCC 20271 strain was sequenced only using a short-read sequencing platform (Illumina MiSeq), which resulted in poor quality of genome assembly (922 contigs, 21 scaffolds with 901 spanned gaps). More importantly, the scaffold with an inconsistent event contained 221 gaps, and some gaps were just located near the location of this inconsistent event. So, we deduced that this inconsistency may well be caused by low-quality assembly and incorrect scaffolding of contigs. Furthermore, we also investigated the genomic synteny of GMU1709 strain with other *Trichosporonaceae* members. The *A. gracile* JCM 10018 and *A. brassicae* JCM 1599 strains from *Apiotrichum* genus exhibited a relatively high genomic collinearity with GMU1709 strain (**Figure S6**), in which the 1:1 homologous block (one-to-one correspondence of homologous region, namely, each reference region matches only one target region) averagely accounted for 87.5% and 81% of the genome, respectively (**Figures S5D–E**); however, the *T. inkin* JCM 9195, *T. faecale* JCM 2941, *C. oleaginosum* ATCC 20508, and *C. daszewskae* JCM 11166 strains from both *Trichosporon* and *Cutaneotrichosporon* genera exhibited low genomic collinearity with GMU1709 strain (**Figure S6**), in which the 1:1 homologous block averagely accounted for 53% and 51.5% of two *Trichosporon* genomes respectively (**Figures S5F–J**) and for 59.5% and 57.5% of two *Cutaneotrichosporon* genomes respectively (**Figures S5H–I**). In addition, compared to intraspecific strains (**Figures S5A–C**), there was a relatively high proportion of ≥ 2:1 homologous block (more-to-one correspondence of homologous region) between GMU1709 and other strains (**Figures S5D–I**). These results support the position that the GMU1709, CICC 1454, and ACCC 20271 strains belong to the *A. mycotoxinivorans*, and also indicate the evolutionary relationship at the genomic level between *A. mycotoxinivorans* and other members in the family *Trichosporonaceae*.

### Genes Putatively Involved in Pathogenicity

**Figure 5:**
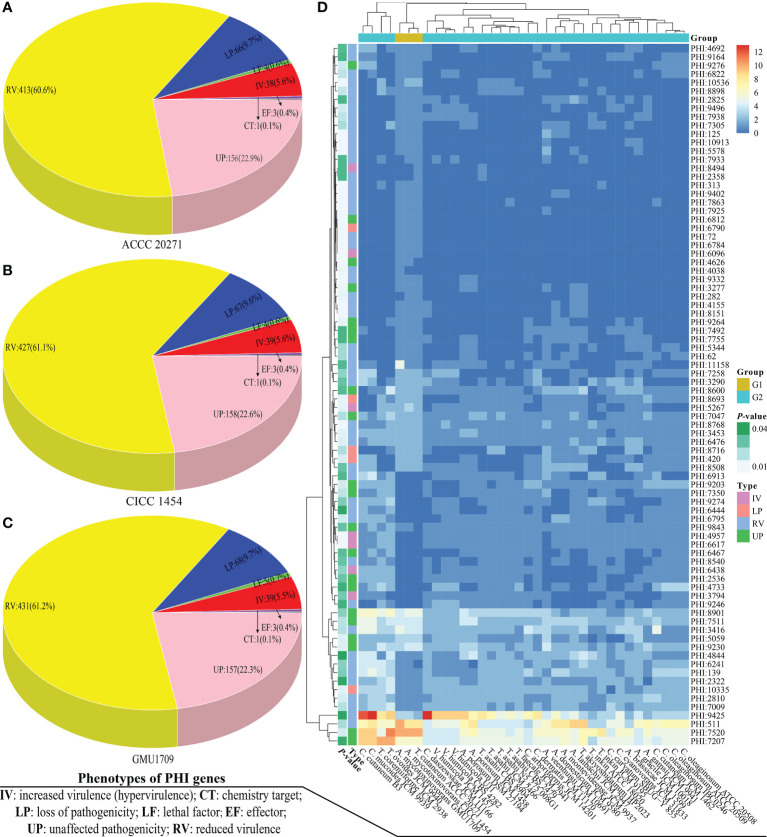
Comparisons of pathogen-host interactions (PHI) annotation results. Number of genes corresponding to different PHI phenotypic terms in *A. mycotoxinivorans* ACCC 20271 **(A)**, CICC 1454 **(B)**, and GMU1709 **(C)** strains. **(D)** Differences (using the Wilcox test) in gene number of PHI-base accessions between *A. mycotoxinivorans* strains (G1 group) and other *Trichosporonaceae* strains (G2 group).

PHI-base is a gene-centric database that catalogs experimentally verified virulence, pathogenicity, and effector genes from different pathogens, in which each gene has a corresponding PHI-base accession number (Urban et al., 2015) and the included pathogens interact with a wide range of hosts. This database is an invaluable resource for discovering pathogenicity-related genes from these emerging opportunistic pathogens, which could be potential intervention targets (Urban et al., 2020). Currently, the PHI-base 4.12 has cataloged 8411 genes and 18 190 interactions (http://www.phi-base.org/releaseNote.htm) to be associated with pathogenicity. *A. mycotoxinivorans* is an emerging opportunistic pathogenic fungus, which is closely related to invasive devices and immunodeficiency (Almeida et al., 2017) and primarily causes pulmonary infections in humans with significant predilection in patients with cystic fibrosis (Marcelo and Farooq, 2018). Using BLAST analysis, we obtained 2149, 2098, and 2068 homologs of PHI genes from GMU1709, CICC 1454, and ACCC 20271 genomes, respectively. By comparing *A. mycotoxinivorans* with other *Trichosporonaceae* species, we found that *A. mycotoxinivorans* strains averagely contained 2105 homologs of PHI genes, which was lower than the average number (2143) of PHI homologs in all strains (**Figure S7**). However, it is noteworthy that *C. mucoides* JCM 9939, *T. coremiiforme* JCM 2938, *T. ovoides* JCM 9940, and *C. cutaneum* B3 contained substantially more homologs of PHI genes than any other strain, thus indicating that these four strains might be closely associated with infection (**Figure S7**). In the PHI-base, nine high-level phenotypic terms (“increased virulence,” “increased virulence (hypervirulence),” “reduced virulence,” “unaffected pathogenicity,” “loss of pathogenicity,” “lethal effector,” “sensitivity to chemical,” and “resistance to chemical”) have been defined to compare the pathogen-host interactions between organisms across the tree of life (Urban et al., 2015). To make the results more reasonable, we retained only putative PHI genes, whose objects of interaction were the “Birds”, “Bony fishes”, “Flies”, “Nematodes”, “Primates”, “Rabbits & hares”, and “Rodents”, because these objects have been used in several cases as model organisms for human diseases. Accordingly, only 704, 699, and 681 homologs of PHI genes (detailed in **Table S3**) were found in the GMU1709, CICC 1454, and ACCC 20271 strains, respectively. Then, we counted these putative PHI genes according to phenotypic terms, and the obtained results indicated that there was approximately the same number of genes with increased virulence (hypervirulence) or lethal factor between environmentally derived strain (ACCC 20271 and CICC 1454; **Figures 5A, B**) and clinically-derived strain (GMU1709; **Figure 5C**). So, these comparisons suggest that the differences between clinical and environmental strains of *A. mycotoxinivorans* may be small.

We further compared the differences of PHI-base entries in the number of genes between *A. mycotoxinivorans* strains and other *Trichosporonaceae* strains *via* the Wilcox test. We deduced that there were 82 PHI-base accessions with a significant difference (*P* < 0.05) (**Table S4** and **Figure 5D**). We further focused on these PHI-base accessions with the phenotype of increased virulence and found three PHI-base accessions with significantly more genes in *A. mycotoxinivorans* strains. More importantly, all three accessions (PHI:6096, PHI:5267, and PHI:8494) were closely linked to pulmonary infections (pneumococcal pneumonia and invasive pulmonary aspergillosis), which is consistent with the phenotype of this opportunistic pathogen. We found that PHI:6096 (Gene locus: Apimy_4244) and PHI: 5267 (Gene locus: Apimy_4588 and Apimy_6176) were present in all three strains; however, PHI:8494 was only present in the ACCC 20271 strain. According to the PHI-base, PHI:5267, PHI:6096, and PHI:8494 correspond to epimerase (UgeB), endopeptidase O (PepO), and phospholipase D (PLD), respectively. Based on above RNA-seq data, we investigated two pathogenic factors (UgeB and PepO) shared by three *A. mycotoxinivorans* strains, and found that genes encoding UgeB had extremely low expression levels with FPKM value ≤0.1, but PepO had a very high gene expression level with FPKM value >4500. Considering that gene expression may vary greatly under different conditions (Guan et al., 2013; Speth et al., 2019), we inferred that both enzymes could be the potentially important pathogenic factors of this emerging opportunistic yeast pathogen.

### Genes Putatively Involved in Antibiotic Resistance

As we previously reported (Peng et al., 2019), the sputum bacterial and fungal cultures of a pediatric patient with pneumonia were positive for *Elizabethkingia anophelis* and *A. mycotoxinivorans* (GMU1709 strain in this study). Considering that *A. mycotoxinivorans* can efficiently degrade tetracycline (Huang et al., 2021), we inferred that the coexistence of *E. anopheles* and *A. mycotoxinivorans* may have improved the antibiotic resistance of *E. anopheles* by inactivating antibiotics. In fact, the interaction between bacteria and fungi is widely known, and the *Candida albicans*-*Pseudomonas aeruginosa* interaction is a well-studied model system (Peleg et al., 2010; Frey-Klett et al., 2011). To comprehensively estimate the possible inactivation of *A. mycotoxinivorans* against antibiotics, we identified all the putative antibiotic resistance genes (126, 122, and 122 genes) from the GMU1709, CICC 1454, and ACCC 20271 strains based on the CARD database (McArthur et al., 2013), respectively, which could be classified into 53 primary ontologies, and deduced that their antibiotic resistance patterns were substantially similar (**Table 3**). Tetracycline resistance had the largest number of ontologies, in which three, six, and 10 primary ontologies were related to antibiotic inactivation, target protection, and efflux, respectively, which is consistent with the findings of Huang et al. (2021). In addition, *A. mycotoxinivorans* might be able to inactivate rifamycin, penam, and cephalosporin. Such inactivation effects are also present to a greater or lesser extent in other species (**Figure S8**). Hence, we further tested the resistance of *E. coli* to tetracycline in the presence/absence of *A. mycotoxinivorans*. As shown in **Figure S9**, when compared to the bacterial survival rate of the group A (live GMU1709 mixed with *E. coli*) (defined as 100%), the bacterial survival rate was (50.3 ± 11.0)% and (52.3 ± 20.1)% for group B (inactivated GMU1709 mixed with *E. coli*) and group C (*E. coli* suspended in saline) respectively after incubation for 2 h (P<0.01), which strongly indicated that *A. mycotoxinivorans* was able to significantly enhanced the resistance of coexisting bacteria to related antibiotics. Moreover, all *A. mycotoxinivorans* strains possessed the same inactivation profiles (**Table 3**, these ARO accessions with antibiotic inactivation). In summary, the coexistence of *A. mycotoxinivorans* with other bacteria may improve the antibiotic resistance of bacteria; hence, this aspect deserves more attention.

**Table 3:** Distribution of putative resistance genes in *A. mycotoxinivorans* strains.

Accession	Drug Class	Resistance Mechanism	Gene Number		
			S1	S2	S3
ARO:3000833	penam, fluoroquinolone antibiotic, macrolide antibiotic, tetracycline antibiotic	antibiotic efflux	13	9	9
ARO:3003942	peptide antibiotic, cephalosporin, penam	antibiotic efflux	11	12	12
ARO:3003950	nitroimidazole antibiotic	antibiotic efflux	10	10	10
ARO:3004574	fluoroquinolone antibiotic	antibiotic efflux	8	8	8
ARO:3002884	rifamycin antibiotic	antibiotic inactivation	6	6	6
ARO:3002522	aminocoumarin antibiotic	antibiotic efflux	5	6	6
ARO:3004036	tetracycline antibiotic	antibiotic efflux	4	4	4
ARO:3000510	mupirocin	antibiotic target alteration	4	4	4
ARO:3002892	tetracycline antibiotic	antibiotic efflux	3	4	4
ARO:3005043	phenicol antibiotic	antibiotic efflux	3	3	3
ARO:3003746	oxazolidinone antibiotic, tetracycline antibiotic, streptogramin antibiotic, macrolide antibiotic, pleuromutilin antibiotic, phenicol antibiotic, lincosamide antibiotic	antibiotic target protection	3	3	3
ARO:3002893	tetracycline antibiotic	antibiotic efflux	3	2	2
ARO:3002947	glycopeptide antibiotic	antibiotic target alteration	3	2	2
ARO:3003980	tetracycline antibiotic	antibiotic efflux	2	2	2
ARO:3000025	fluoroquinolone antibiotic	antibiotic efflux	2	2	2
ARO:3000193	tetracycline antibiotic	antibiotic target protection	2	2	2
ARO:3002812	phenicol antibiotic	antibiotic efflux	2	2	2
ARO:3004611	cephalosporin, penam	antibiotic inactivation	2	2	2
ARO:3002943	glycopeptide antibiotic	antibiotic target alteration	2	2	2
ARO:3000421	fluoroquinolone antibiotic	antibiotic efflux	2	2	2
ARO:3003986	pleuromutilin antibiotic	antibiotic efflux	2	2	2
ARO:3002699	phenicol antibiotic	antibiotic efflux	2	2	2
ARO:3005056	tetracycline antibiotic	antibiotic inactivation	2	2	2
ARO:3002945	glycopeptide antibiotic	antibiotic target alteration	2	2	2
ARO:3000195	tetracycline antibiotic	antibiotic target protection	2	2	2
ARO:3004033	tetracycline antibiotic	antibiotic efflux	2	1	1
ARO:3002942	glycopeptide antibiotic	antibiotic target alteration	1	1	1
ARO:3005057	tetracycline antibiotic	antibiotic inactivation	1	1	1
ARO:3002881	lincosamide antibiotic, phenicol antibiotic, streptogramin antibiotic, pleuromutilin antibiotic, tetracycline antibiotic, oxazolidinone antibiotic, macrolide antibiotic	antibiotic target protection	1	1	1
ARO:3000838	disinfecting agents and intercalating dyes, fluoroquinolone antibiotic, acridine dye	antibiotic efflux	1	1	1
ARO:3005063	peptide antibiotic	antibiotic target alteration, antibiotic efflux	1	1	1
ARO:3002883	rifamycin antibiotic	antibiotic inactivation	1	1	1
ARO:3000183	tetracycline antibiotic	antibiotic efflux	1	1	1
ARO:3002985	peptide antibiotic	antibiotic target alteration	1	1	1
ARO:3001329	fosfomycin	antibiotic efflux	1	1	1
ARO:3004476	streptogramin antibiotic, lincosamide antibiotic, pleuromutilin antibiotic, oxazolidinone antibiotic, phenicol antibiotic, tetracycline antibiotic, macrolide antibiotic	antibiotic target protection	1	1	1
ARO:3000572	tetracycline antibiotic	antibiotic efflux	1	1	1
ARO:3001313	elfamycin antibiotic	antibiotic efflux	1	1	1
ARO:3003801	bicyclomycin	antibiotic efflux	1	1	1
ARO:3003749	tetracycline antibiotic, lincosamide antibiotic, pleuromutilin antibiotic, phenicol antibiotic, streptogramin antibiotic, oxazolidinone antibiotic, macrolide antibiotic	antibiotic target protection	1	1	1
ARO:3002882	lincosamide antibiotic	antibiotic efflux	1	1	1
ARO:3000501	rifamycin antibiotic	antibiotic target alteration, antibiotic target replacement	1	1	1
ARO:3003953	disinfecting agents and intercalating dyes, acridine dye, fluoroquinolone antibiotic	antibiotic efflux	1	1	1
ARO:3005345	diaminopyrimidine antibiotic	antibiotic target replacement	1	1	1
ARO:3004361	sulfonamide antibiotic	antibiotic target replacement	1	1	1
ARO:3000175	tetracycline antibiotic	antibiotic efflux	1	1	1
ARO:3004613	tetracycline antibiotic	antibiotic inactivation	1	1	1
ARO:3003046	fluoroquinolone antibiotic	antibiotic efflux	1	1	1
ARO:3000822	fluoroquinolone antibiotic	antibiotic efflux	1	0	0
ARO:3002928	glycopeptide antibiotic	antibiotic target alteration	1	0	0
ARO:3002944	glycopeptide antibiotic	antibiotic target alteration	0	1	1
ARO:3000549	tetracycline antibiotic, glycylcycline	antibiotic efflux	0	1	0
ARO:3002929	glycopeptide antibiotic	antibiotic target alteration	0	0	1

The S1, S2 and S3 represent the strains GMU1709, CICC 1454, and ACCC 20271, respectively.

In our previous study (Peng et al., 2019), the sensitivity of the GMU1709 strain to antifungal drugs was determined using the commercial method ATB FUNGUS 3, and our results indicated that the *A. mycotoxinivorans* GMU1709 strain is sensitive to the antifungal drugs, including several triazoles, flucytosine, and amphotericin B. Notably, amphotericin B also exhibits high efficacy in treating *A. mycotoxinivorans* GMU1709 infection in our previous study (Peng et al., 2019), which is in contrast with previous clinical reports (Hickey et al., 2009; Goldenberger et al., 2016). Here, we further detected the genes possibly involved in antifungal drug resistance in the GMU1709, CICC 1454, and ACCC 20271 strains based on MARDy (Nash et al., 2018). Consistent with our previous experimental results obtained from the antifungal susceptibility testing for the GMU1709 strain, we did not detect any gene or genetic events associated with antifungal drug resistance. These results also indicated that all three strains were sensitive to these antifungal drugs, which is consistent with the findings of Almeida et al. (2017) that clinical and environmental isolates of *A. mycotoxinivorans* had similar spectral profiles in genetic, proteomic diversity, and antibiotic resistance, based on antifungal susceptibility tests.

## Discussion

*A. mycotoxinivorans* is a versatile yeast that is not only able to efficiently detoxify mycotoxins (Molnar et al., 2004) and degrade tetracycline, but also turn organic wastes (such as oily waste and carbohydrates) or pollutants into microbial lipids (Sagia et al., 2020), and produce bioemulsifiers that could be used to bioemulsify oils (Domingues et al., 2017). Hence, it has important application value for realizing sustainable energy development and eliminating environmental and food contamination. More notably, this yeast is a potentially fatal cause of disseminated and localized infections in immunocompromised patients (Hickey et al., 2009). In a previous study, we isolated an *A. mycotoxinivorans* strain from the sputum of a pediatric patient with congenital ventricular septal defect and pulmonary *E. anopheles* infection (Peng et al., 2019). Accordingly, in addition to helping us better understand its genomic signatures, deciphering the genome of *A. mycotoxinivorans* also provides clear genetic information for promoting its potential applications and developing prevention and control strategies against infection. Here, we adopted the Illumina and Nanopore platforms, completed the genome assembly and annotation of *A. mycotoxinivorans* for the first time, obtained a genome assembly with a contig N50 of 7.4 MB, comprising seven ungapped sequences. Six out of seven genomic sequences were anchored by Hi-C data to six chromosomes (**Figure 1A**), and the remaining genomic sequence was the mitochondrial sequence. Together with the evaluation of genomic integrity and fidelity (**Tables S2**, **S5**), these results indicate a high-quality and complete genome. Combined with transcriptome data, 8066 protein-coding genes were identified, and the structural features of nuclear genes (such as exon number and exon length) were consistent with those of other species of the same genus. Hence, the high-quality genome of GMU1709 and its relevant information provide an important data source for studying the characteristics of *A. mycotoxinivorans*.

Although the basidiomycetous yeast is conventionally classified according to morphological characteristics, its phenotypic characteristics do not reflect phylogeny in many cases (Liu et al., 2015). Consequently, the phylogenetic analysis of universally conserved proteins or genes (especially rRNA genes) has gradually become a powerful tool for the classification of basidiomycetous yeast. However, phylogenetic analyses of single or multiple genes exhibit only low resolution of phylogenetic relationships and cannot provide sufficient information to resolve deep branches (Fitz-Gibbon and House, 1999). Furthermore, different evolutionary rates or horizontal gene transfers would distort the topology of the phylogenetic tree constructed based on a single gene or a few genes, which may incorrectly influence their phylogenetic relationships. Aliyu et al. (2020) constructed a phylogenetic tree of 33 strains in the *Trichosporonaceae* family based on 405 orthologous proteins and identified two distinct misidentifications in which *T. akiyoshidainum* HP2023 and *C. cutaneum* ACCC 20271 were reassigned to *Apiotrichum*. In a previous study (Peng et al., 2019), we identified the GMU1709 strain as *A. mycotoxinivorans via* a 26S rDNA-based phylogenetic relationship, including biochemical and morphological characteristics. In this study, we performed a whole genome-based phylogenetic analysis based on multi-genome alignments for four major genera of *Trichosporonaceae*. In our results, the formerly named *T. cutaneum* ACCC 20271 strain belonged to *A. mycotoxinivorans* (**Figure 2**), and the genome collinearity analysis (**Figure 4**), gene pattern clustering, and Venn diagrams of shared relationships between genus or/and strains (**Figure 3**) all supported this finding. In addition, the HP2023 strain was reassigned to *A. akiyoshidainum* based on our results and previous reports (Aliyu et al., 2020). Therefore, our genome-based phylogenetic analysis further clarified the evolutionary relationship between *A. mycotoxinivorans* and other *Trichosporonaceae* members.

Recently, opportunistic fungal infections have become more common, owing to the rapid increase in the number of immunocompromised patients, primarily caused by immunosuppressive therapies (such as organ transplants, cytotoxic chemotherapies, and the use of intravenous catheters) or the human immunodeficiency virus (Pérez-Torrado and Querol, 2016). *A. mycotoxinivorans* has also been recognized as an emerging opportunistic yeast pathogen that causes infections in immunocompromised patients (do Espírito Santo et al., 2020). In this study, differences in pathogenic genetic basis between clinical and non-clinical strains of *A. mycotoxinivorans* seem small to non-existent, which is consistent with the findings of Pfaller (2015), who reported that any fungus can cause infection in a sufficiently immunocompromised host. For example, although *Saccharomyces cerevisiae* has a well-documented history of safe use in food (Sewalt et al., 2016), its colonization of the human body, whether long-lasting or transient, may occasionally result in infections (Anoop et al., 2015). *A. mycotoxinivorans* primarily causes pulmonary infection in immunocompromised humans, with a higher propensity for patients with cystic fibrosis (Marcelo and Farooq, 2018). *Via* comparative analysis with other strains of the *Trichosporonaceae* family, in *A. mycotoxinivorans* strains, we deduced that there are three PHI-base genes (including the PHI:5267: UgeB, PHI:6096: PepO, and PHI:8494: PLD) with a phenotype of increased virulence (hypervirulence) and significantly more genes, which are closely related to pulmonary infections. PepO is a newly discovered virulence protein correlated with host cell adhesion and invasion (Zhang et al., 2016). In 36 strains from four genera of the *Trichosporonaceae* family, PepO was determined to solely exist in three *A. mycotoxinivorans* strains and was predicted to be a transmembrane protein (a portion of the PepO protein sequence may be extracellular), which could play an important role in the pathogen invasion of host cells mediated by host cell adhesion and the immune evasion mediated by binding to plasminogen and fibronectin. UgeB encodes the epimerase required for the synthesis of N-acetyl-galactosamine and subsequent production of galactosaminogalactan (Henriet et al., 2016). Animal experiments have demonstrated that the overexpression of UgeB increases the amount of cell wall-bound galactosaminogalactan, thereby enhancing the adherence and virulence of the pathogen (Lee et al., 2015). We determined that two or three genes encoded UgeB in *A. mycotoxinivorans* strains, which is significantly higher than almost all strains of other species (containing only zero or one gene; **Table S4**). According to the report by Lee et al. (2015), high amounts of cell wall-bound galactosaminogalactan may increase its virulence by mediating resistance to NADPH oxidase-dependent neutrophil extracellular traps. PLD is an essential enzyme responsible for producing the lipid second messenger phosphatidic acid, which is involved in several fundamental cellular processes, including actin cytoskeleton remodeling, membrane trafficking, cell survival, and proliferation (Oude Weernink et al., 2007). This enzyme is an important virulence factor for pathogen infections and can enhance the production of ROS by increasing the expression level of histone deacetylase (a regulator of inflammatory responses and ROS production) involved in immunomodulation during infection (Yu and Huo, 2018). Genes encoding PLD are solely present in the non-clinical ACCC 20271 strain, and a previous study has demonstrated that it can cause invasive pulmonary aspergillosis. This gene might be obtained *via* horizontal gene transfer (Van Etten and Bhattacharya, 2020), which may further enhance its virulence. From above discussion, it is clear that no evidence exists to support that clinical strain had stronger pathogenicity than non-clinical strain, and above three proteins may well be directly related to the opportunistic infections of *A. mycotoxinivorans*.

It has been reported that fungi and bacteria live together in a wide variety of environments and engage in several types of interactions that lead to their behavioral shifts (Deveau et al., 2018). In our previous study, we isolated and identified two pathogens (the *A. mycotoxinivorans* GMU1709 strain and an *E. anopheles* strain) from sputum specimens collected with bronchofiberscopes (Peng et al., 2019). Considering that *A. mycotoxinivorans* can degrade antibiotics, including tetracycline (Huang et al., 2021), *A. mycotoxinivorans* could help pathogenic bacteria survive by inactivating antibiotics. We examined the antibiotic resistance genes from three *A. mycotoxinivorans* strains and determined that they all contained putative genes responsible for inactivating tetracycline, rifamycin, penam, and cephalosporin. Therefore, *A. mycotoxinivorans*, when living with bacteria, is substantially likely to increase the resistance of the surrounding bacteria to these antibiotics, thus making antibiotic therapy ineffective. In addition, our previous experimental analysis indicates that the GMU1709 strain is not resistant to antifungal drugs (Peng et al., 2019), which is consistent with the fact that no gene or mutation associated with antifungal drug resistance was identified in any of three *A. mycotoxinivorans* strains (including the GMU1709 strain) in this study. This further shows that there is no difference between clinical and non-clinical strains in antifungal drug resistance, which is consistent with the findings of Almeida et al. (2017). Hence, these results indicate that *A. mycotoxinivorans* can interact with pathogenic bacteria to resist antibiotic treatment, and there is also no significant genetic difference in drug resistance between clinical and non-clinical strains.

## Conclusion

For the first time, we reported a clinically-derived *A. mycotoxinivorans* (GMU1709) genome with high-quality annotation, using Illumina, Nanopore, and Hi-C technologies. The assembly exhibited a higher level of completeness and genome quality than other *Apiotrichum* genomes. Comparative genomic and phylogenomic analyses supported the classification of the formerly named *T. cutaneum* ACCC 20271 strain within the *A. mycotoxinivorans* species and provided further evidence for the evolutionary relationships of *A. mycotoxinivorans* and other *Trichosporonaceae* members. We found no obvious genetic difference in drug resistance and pathogenicity between clinical and non-clinical strains of *A. mycotoxinivorans*. More importantly, we identified the putative virulence factors and drug resistance genes of *A. mycotoxinivorans*, which could be potential targets for the further research and therapeutic intervention of such opportunistic infections. In conclusion, this study lays a solid foundation for understanding the phylogenetic relationship of *Trichosporonaceae*, promoting the potential application of *A. mycotoxinivorans*, and developing disease prevention and control strategies.

## Data Availability Statement

The datasets presented in this study can be found in online repositories. The names of the repository/repositories and accession number(s) can be found in the article/**Supplementary Material**.

## Author Contributions

Z-KY and LP conceived the project. LP, Z-KY, C-FL, HW, HJ, X-YD, L-TZ, YX, and CD prepared the strain samples and performed sequence analyses. Z-KY conducted the bioinformatics analyses. C-FL performed the additional experiments. Z-KY prepared the manuscript. LP, C-FL, HW, HJ, X-YD, and YX participated in discussions and provided suggestions. All authors read and approved the final manuscript.

## Funding

This study is funded by the Guangzhou Key Laboratory Fund (201905010004), and Science and Technology Program of Guangzhou (No. 202002030419).

## Conflict of Interest

The authors declare that the research was conducted in the absence of any commercial or financial relationships that could be construed as a potential conflict of interest.

## Publisher’s Note

All claims expressed in this article are solely those of the authors and do not necessarily represent those of their affiliated organizations, or those of the publisher, the editors and the reviewers. Any product that may be evaluated in this article, or claim that may be made by its manufacturer, is not guaranteed or endorsed by the publisher.

## References

[B1] Aliyu H. Gorte O. De Maayer P. Neumann A. Ochsenreither K. (2020). Genomic Insights Into the Lifestyles, Functional Capacities and Oleagenicity of Members of the Fungal Family Trichosporonaceae. Sci. Rep. 10, 1–12. doi: 10.1038/s41598-020-59672-2 32066798 PMC7026411

[B2] Almeida J. N. D. Jr. Francisco E. C. Barberino M. G. M. da Silva Filho L. V. R. F. Brandão O. M. Colombo A. L.. (2017). Emergence of Trichosporon Mycotoxinivorans (*Apiotrichum Mycotoxinivorans*) Invasive Infections in Latin America. Mem I Oswaldo Cruz 112, 719–722. doi: 10.1590/0074-02760170011 PMC5607521 28954000

[B3] Altschul S. F. Madden T. L. Schäffer A. A. Zhang J. Zhang Z. Miller W.. (1997). Gapped BLAST and PSI-BLAST: A New Generation of Protein Database Search Programs. Nucleic Acids Res. 25, 3389–3402. doi: 10.1093/nar/25.17.3389 9254694 PMC146917

[B4] Angiuoli S. V. Salzberg S. L. (2010). Mugsy: Fast Multiple Alignment of Closely Related Whole Genomes. Bioinformatics 27, 334–342. doi: 10.1093/bioinformatics/btq665 21148543 PMC3031037

[B5] Anoop V. Rotaru S. Shwed P. S. Tayabali A. F. Arvanitakis G. (2015). Review of Current Methods for Characterizing Virulence and Pathogenicity Potential of Industrial Saccharomyces Cerevisiae Strains Towards Humans. FEMS Yeast Res. 15, fov057. doi: 10.1093/femsyr/fov057 26195617

[B6] Birney E. Clamp M. Durbin R. (2004). GeneWise and Genomewise. Genome Res. 14, 988–995. doi: 10.1101/gr.1865504 15123596 PMC479130

[B7] Blanco E. Parra G. Guigó R. (2007). Using Geneid to Identify Genes. Curr. Protoc. Bioinf. 18, 4.3.1–4.3.28. doi: 10.1002/0471250953.bi0403s18 18428791

[B8] Bliznina A. Masunaga A. Mansfield M. J. Tan Y. Liu A. W. West C.. (2021). Telomere-To-Telomere Assembly of the Genome of an Individual Oikopleura Dioica From Okinawa Using Nanopore-Based Sequencing. BMC Genomics 22, 222. doi: 10.1186/s12864-021-07512-6 33781200 PMC8008620

[B9] Buchfink B. Xie C. Huson D. H. (2015). Fast and Sensitive Protein Alignment Using DIAMOND. Nat. Methods 12, 59–60. doi: 10.1038/nmeth.3176 25402007

[B10] Burge C. Karlin S. (1997). Prediction of Complete Gene Structures in Human Genomic DNA. J. Mol. Biol. 268, 78–94. doi: 10.1006/jmbi.1997.0951 9149143

[B11] Burton J. N. Adey A. Patwardhan R. P. Qiu R. Kitzman J. O. Shendure J. (2013). Chromosome-Scale Scaffolding of *De Novo* Genome Assemblies Based on Chromatin Interactions. Nat. Biotechnol. 31, 1119–1125. doi: 10.1038/nbt.2727 24185095 PMC4117202

[B12] Close D. Ojumu J. (2016). Draft Genome Sequence of the Oleaginous Yeast Cryptococcus Curvatus ATCC 20509. Genome Announcements 4, e01235–e01216. doi: 10.1128/genomeA.01235-16 27811111 PMC5095481

[B13] Dabas Y. Xess I. Kale P. (2017). Molecular and Antifungal Susceptibility Study on Trichosporonemia and Emergence of Trichosporon Mycotoxinivorans as a Bloodstream Pathogen. Med. Mycol 55, 518–527. doi: 10.1093/mmy/myw100 27816903

[B14] Deng Y. Y. Li J. Q. Wu S. F. Zhu Y. P. Chen Y. W. He F. C. (2006). Integrated Nr Database in Protein Annotation System and Its Localization. Comput. Eng. 32, 71–72.

[B15] Deveau A. Bonito G. Uehling J. Paoletti M. Becker M. Bindschedler S.. (2018). Bacterial-Fungal Interactions: Ecology, Mechanisms and Challenges. FEMS Microbiol. Rev. 42, 335–352. doi: 10.1093/femsre/fuy008 29471481

[B16] do Espírito Santo E. P. T. Monteiro R. C. da Costa A. R. F. Marques-Da-Silva S. H. (2020). Molecular Identification, Genotyping, Phenotyping, and Antifungal Susceptibilities of Medically Important Trichosporon, Apiotrichum, and Cutaneotrichosporon Species. Mycopathologia 185, 307–317. doi: 10.1007/s11046-019-00407-x 31776790

[B17] Domingues V. S. Monteiro A. S. Ferreira G. F. Santos V. L. (2017). Solid Flocculation and Emulsifying Activities of the Lipopolysaccharide Produced by Trichosporon Mycotoxinivorans CLA2. Appl. Biochem. Biotech. 182, 367–381. doi: 10.1007/s12010-016-2332-0 27917440

[B18] Duan Z. Andronescu M. Schutz K. Lee C. Shendure J. Fields S.. (2012). A Genome-Wide 3C-Method for Characterizing the Three-Dimensional Architectures of Genomes. Methods (San Diego Calif) 58, 277–288. doi: 10.1016/j.ymeth.2012.06.018 PMC3477625 22776363

[B19] Edgar R. C. (2004). MUSCLE: Multiple Sequence Alignment With High Accuracy and High Throughput. Nucleic Acids Res. 32, 1792–1797. doi: 10.1093/nar/gkh340 15034147 PMC390337

[B20] Fitz-Gibbon S. T. House C. H. (1999). Whole Genome-Based Phylogenetic Analysis of Free-Living Microorganisms. Nucleic Acids Res. 27, 4218–4222. doi: 10.1093/nar/27.21.4218 10518613 PMC148696

[B21] Frey-Klett P. Burlinson P. Deveau A. Barret M. Tarkka M. Sarniguet A. (2011). Bacterial-Fungal Interactions: Hyphens Between Agricultural, Clinical, Environmental, and Food Microbiologists. Microbiol. Mol. Biol. Rev. 75, 583–609. doi: 10.1128/MMBR.00020-11 22126995 PMC3232736

[B22] Gil N. M. B. Pajot H. F. Soro M. D. M. R. de Figueroa L. I. C. Kurth D. (2018). Genome-Wide Overview of Trichosporon Akiyoshidainum HP-2023, New Insights Into Its Mechanism of Dye Discoloration. 3. Biotech 8, 440. doi: 10.1007/s13205-018-1465-y PMC6173679 30306009

[B23] Goldenberger D. Hinić V. Prince S. S. Tamm M. Balestra A. M. Hohler D.. (2016). A Case Report of a Cystic Fibrosis Patient With Repeated Isolation of Trichosporon Mycotoxinivorans Identified by a Novel Short-Extraction Method. BMC Infect. Dis. 16, 601. doi: 10.1186/s12879-016-1910-7 27782810 PMC5078883

[B24] Gorte O. Aliyu H. Neumann A. Ochsenreither K. (2019). Draft Genome Sequence of the Oleaginous Yeast Apiotrichum Porosum (Syn. *Trichosporon Porosum*) DSM 27194. J. Genomics 7, 11–13. doi: 10.7150/jgen.32210 30820256 PMC6389497

[B25] Guan Y. Dunham M. J. Troyanskaya O. G. Caudy A. A. (2013). Comparative Gene Expression Between Two Yeast Species. BMC Genomics 14, 33. doi: 10.1186/1471-2164-14-33 23324262 PMC3556494

[B26] Haas B. J. Papanicolaou A. Yassour M. Grabherr M. Blood P. D. Bowden J.. (2013). *De Novo* Transcript Sequence Reconstruction From RNA-Seq Using the Trinity Platform for Reference Generation and Analysis. Nat. Protoc. 8, 1494–1512. doi: 10.1038/nprot.2013.084 23845962 PMC3875132

[B27] Haas B. J. Salzberg S. L. Zhu W. Pertea M. Allen J. E. Orvis J.. (2008). Automated Eukaryotic Gene Structure Annotation Using EVidenceModeler and the Program to Assemble Spliced Alignments. Genome Biol. 9, R7–R7. doi: 10.1186/gb-2008-9-1-r7 18190707 PMC2395244

[B28] Henriet S. S. van de Sande W. W. Lee M. J. Simonetti E. Momany M. Verweij P. E.. (2016). Decreased Cell Wall Galactosaminogalactan in Aspergillus Nidulans Mediates Dysregulated Inflammation in the Chronic Granulomatous Disease Host. J. Interf. Cytok. Res. 36, 488–498. doi: 10.1089/jir.2015.0095 27142572

[B29] Hickey P. W. Sutton D. A. Fothergill A. W. Rinaldi M. G. Wickes B. L. Schmidt H. J.. (2009). Trichosporon Mycotoxinivorans, a Novel Respiratory Pathogen in Patients With Cystic Fibrosis. J. Clin. Microbiol. 47, 3091–3097. doi: 10.1128/JCM.00460-09 19656976 PMC2756937

[B30] Hirschi S. Letscher-Bru V. Pottecher J. Lannes B. Jeung M. Y. Degot T.. (2012). Disseminated Trichosporon Mycotoxinivorans, Aspergillus Fumigatus, and Scedosporium Apiospermum Coinfection After Lung and Liver Transplantation in a Cystic Fibrosis Patient. J. Clin. Microbiol. 50, 4168–4170. doi: 10.1128/JCM.01928-12 23035187 PMC3503015

[B31] Huang X. Zhang X. Huang Y. Xu X. (2021). Optimization of Media Composition for Enhancing Tetracycline Degradation by Trichosporon Mycotoxinivorans XPY-10 Using Response Surface Methodology. Environ. Technol. 42, 4279–4285. doi: 10.1080/09593330.2020.1754472 32270748

[B32] Jia B. Raphenya A. R. Alcock B. Waglechner N. Guo P. Tsang K. K.. (2017). CARD 2017: Expansion and Model-Centric Curation of the Comprehensive Antibiotic Resistance Database. Nucleic Acids Res. 45, D566–D573. doi: 10.1093/nar/gkw1004 27789705 PMC5210516

[B33] Keilwagen J. Wenk M. Erickson J. L. Schattat M. H. Grau J. Hartung F. (2016). Using Intron Position Conservation for Homology-Based Gene Prediction. Nucleic Acids Res. 44, e89–e89. doi: 10.1093/nar/gkw092 26893356 PMC4872089

[B34] Khalel A. S. Khaled J. M. Saleh A. K. (2012). Enzymatic Activity and Some Molecular Properties of Trichosporon Mycotoxinovorans Yeast and Their Effect on Liver Function in Mice. Afr J. Microbiol. Res. 6, 2567–2573. doi: 10.5897/AJMR12.141

[B35] Kim J. S. Seo S. G. Jun B. K. Kim J. W. Kim S. H. (2010). Simple and Reliable DNA Extraction Method for the Dark Pigmented Fungus, Cercospora Sojina. Plant Pathol. J. 26, 289–292. doi: 10.5423/PPJ.2010.26.3.289

[B36] Koren S. Walenz B. P. Berlin K. Miller J. R. Bergman N. H. Phillippy A. M. (2017). Canu: Scalable and Accurate Long-Read Assembly *via* Adaptive K-Mer Weighting and Repeat Separation. Genome Res. 27, 722–736. doi: 10.1101/gr.215087.116 28298431 PMC5411767

[B37] Korf I. (2004). Gene Finding in Novel Genomes. BMC Bioinf. 5, 59–59. doi: 10.1186/1471-2105-5-59 PMC421630 15144565

[B38] Krogh A. Larsson B.È Von Heijne G. Sonnhammer E. L. (2001). Predicting Transmembrane Protein Topology With a Hidden Markov Model: Application to Complete Genomes. J. Mol. Biol. 305, 567–580. doi: 10.1006/jmbi.2000.4315 11152613

[B39] Lee M. J. Liu H. Barker B. M. Snarr B. D. Gravelat F. N. Al Abdallah Q.. (2015). The Fungal Exopolysaccharide Galactosaminogalactan Mediates Virulence by Enhancing Resistance to Neutrophil Extracellular Traps. PloS Pathog. 11, e1005187. doi: 10.1371/journal.ppat.1005187 26492565 PMC4619649

[B40] Letunic I. Bork P. (2019). Interactive Tree Of Life (iTOL) V4: Recent Updates and New Developments. Nucleic Acids Res. 47, W256–W259. doi: 10.1093/nar/gkz239 30931475 PMC6602468

[B41] Li H. Handsaker B. Wysoker A. Fennell T. Ruan J. Homer N.. (2009). The Sequence Alignment/Map Format and SAMtools. Bioinformatics 25, 2078–2079. doi: 10.1093/bioinformatics/btp352 19505943 PMC2723002

[B42] Li L. Stoeckert C. J. Roos D. S. (2003). OrthoMCL: Identification of Ortholog Groups for Eukaryotic Genomes. Genome Res. 13, 2178–2189. doi: 10.1101/gr.1224503 12952885 PMC403725

[B43] Liu X. Z. Wang Q. M. Göker M. Groenewald M. Kachalkin A. V. Lumbsch H. T.. (2015). Towards an Integrated Phylogenetic Classification of the Tremellomycetes. Stud. Mycol. 81, 85–147. doi: 10.1016/j.simyco.2015.07.001 26955199 PMC4777781

[B44] Lowe T. M. Eddy S. R. (1997). Trnascan-SE: A Program for Improved Detection of Transfer RNA Genes in Genomic Sequence. Nucleic Acids Res. 25, 955–964. doi: 10.1093/nar/25.5.955 9023104 PMC146525

[B45] Majoros W. H. Pertea M. Salzberg S. L. (2004). TigrScan and GlimmerHMM: Two Open Source Ab Initio Eukaryotic Gene-Finders. Bioinformatics 20, 2878–2879. doi: 10.1093/bioinformatics/bth315 15145805

[B46] Marcelo C. Farooq F. (2018). A Trich-Y Fungus: A Unique Presentation of Disseminated Trichosporon Mycotoxinivorans Infection. J. Pioneer Med. Sci. 8, 37–40.

[B47] McArthur A. G. Waglechner N. Nizam F. Yan A. Azad M. A. Baylay A. J.. (2013). The Comprehensive Antibiotic Resistance Database. Antimicrob. Agents Ch. 57, 3348–3357. doi: 10.1128/AAC.00419-13 PMC3697360 23650175

[B48] Molnar O. Schatzmayr G. Fuchs E. Prillinger H. (2004). Trichosporon Mycotoxinivorans Sp. Nov., A New Yeast Species Useful in Biological Detoxification of Various Mycotoxins. Syst. Appl. Microbiol. 27, 661–671. doi: 10.1078/0723202042369947 15612623

[B49] Nash A. Sewell T. Farrer R. A. Abdolrasouli A. Shelton J. M. Fisher M. C.. (2018). MARDy: Mycology Antifungal Resistance Database. Bioinformatics 34, 3233–3234. doi: 10.1093/bioinformatics/bty321 29897419 PMC6137992

[B50] Nawrocki E. P. Burge S. W. Bateman A. Daub J. Eberhardt R. Y. Eddy S. R.. (2014). Rfam 12.0: Updates to the RNA Families Database. Nucleic Acids Res. 43, D130–D137. doi: 10.1093/nar/gku1063 25392425 PMC4383904

[B51] Nawrocki E. P. Eddy S. R. (2013). Infernal 1.1: 100-Fold Faster RNA Homology Searches. Bioinformatics 29, 2933–2935. doi: 10.1093/bioinformatics/btt509 24008419 PMC3810854

[B52] Oude Weernink P. A. López de Jesús M. Schmidt M. (2007). Phospholipase D Signaling: Orchestration by PIP2 and Small GTPases. N-S Arch. Pharmacol. 374, 399–411. doi: 10.1007/s00210-007-0131-4 PMC2020506 17245604

[B53] Peleg A. Y. Hogan D. A. Mylonakis E. (2010). Medically Important Bacterial–Fungal Interactions. Nat. Rev. Microbiol. 8, 340–349. doi: 10.1038/nrmicro2313 20348933

[B54] Peng L. Jiang Y. Q. Jiang G. M. Ou J. Y. Zeng L. T. Zhang H. H.. (2019). Molecular Identification and Biological Characteristic Analysis of an Apiotrichum Mycotoxinivorans (Formerly *Trichosporon Mycotoxinivorans*) Strain Isolated From Sputum Specimens of a Pediatric Patient With Pneumonia. J. Mycol. Med. 29, 120–126. doi: 10.1016/j.mycmed.2019.01.010 30898449

[B55] Pérez-Torrado R. Querol A. (2016). Opportunistic Strains of Saccharomyces Cerevisiae: A Potential Risk Sold in Food Products. Front. Microbiol. 6, 1522. doi: 10.3389/fmicb.2015.01522 26779173 PMC4705302

[B56] Pertea M. Kim D. Pertea G. M. Leek J. T. Salzberg S. L. (2016). Transcript-Level Expression Analysis of RNA-Seq Experiments With HISAT, StringTie and Ballgown. Nat. Protoc. 11, 1650–1667. doi: 10.1038/nprot.2016.095 27560171 PMC5032908

[B57] Peska V. Garcia S. (2020). Origin, Diversity, and Evolution of Telomere Sequences in Plants. Front. Plant Sci. 11, 117. doi: 10.3389/fpls.2020.00117 32153618 PMC7046594

[B58] Petersen T. N. Brunak S. Von Heijne G. Nielsen H. (2011). SignalP 4.0: Discriminating Signal Peptides From Transmembrane Regions. Nat. Methods 8, 785–786. doi: 10.1038/nmeth.1701 21959131

[B59] Pfaller M. A. (2015). Invasive Fungal Infections and Approaches to Their Diagnosis. Method Microbiol. 42, 219–287. doi: 10.1016/bs.mim.2015.05.002

[B60] Rahnama M. Wang B. Dostart J. Novikova O. Yackzan D. Yackzan A.. (2021). Telomere Roles in Fungal Genome Evolution and Adaptation. Front. Genet. 12, 676751. doi: 10.3389/fgene.2021.676751 34434216 PMC8381367

[B61] Rodríguez A. Burgon J. D. Lyra M. Irisarri I. Baurain D. Blaustein L.. (2017). Inferring the Shallow Phylogeny of True Salamanders (*Salamandra*) by Multiple Phylogenomic Approaches. Mol. Phylogenet. Evol. 115, 16–26. doi: 10.1016/j.ympev.2017.07.009 28716741

[B62] Ruan J. Li H. (2020). Fast and Accurate Long-Read Assembly With Wtdbg2. Nat. Methods 17, 155–158. doi: 10.1038/s41592-019-0669-3 31819265 PMC7004874

[B63] Sadamatsu H. Takahashi K. Tashiro H. Ogusu S. Haraguchi T. Nakashima C.. (2020). A Rare Case of Trichosporon Mycotoxinivorans and Cryptococcus Neoformans Co-Infection in Lung. J. Infect. Chemother. 26, 838–842. doi: 10.1016/j.jiac.2020.03.002 32249160

[B64] Sagia S. Sharma A. Singh S. Chaturvedi S. Nain P. K. S. Nain L. (2020). Single Cell Oil Production by a Novel Yeast Trichosporon Mycotoxinivorans for Complete and Ecofriendly Valorization of Paddy Straw. Electron J. Biotechn. 44, 60–68. doi: 10.1016/j.ejbt.2020.01.009

[B65] Schatzmayr G. Heidler D. Fuchs E. Nitsch S. Mohnl M. Täubel M.. (2003). Investigation of Different Yeast Strains for the Detoxification of Ochratoxin A. Mycotoxin Res. 19, 124–128. doi: 10.1007/BF02942950 23604763

[B66] Servant N. Varoquaux N. Lajoie B. R. Viara E. Chen C. J. Vert J. P.. (2015). HiC-Pro: An Optimized and Flexible Pipeline for Hi-C Data Processing. Genome Biol. 16, 259. doi: 10.1186/s13059-015-0831-x 26619908 PMC4665391

[B67] Sewalt V. Shanahan D. Gregg L. La Marta J. Carrillo R. (2016). The Generally Recognized as Safe (GRAS) Process for Industrial Microbial Enzymes. Ind. Biotechnol. 12, 295–302. doi: 10.1089/ind.2016.0011

[B68] Shah A. V. McColley S. A. Weil D. Zheng X. (2014). Trichosporon Mycotoxinivorans Infection in Patients With Cystic Fibrosis. J. Clin. Microbiol. 52, 2242–2244. doi: 10.1128/JCM.03309-13 24648553 PMC4042814

[B69] She R. Chu J. S. C. Wang K. Pei J. Chen N. (2009). GenBlastA: Enabling BLAST to Identify Homologous Gene Sequences. Genome Res. 19, 143–149. doi: 10.1101/gr.082081.108 18838612 PMC2612959

[B70] Simão F. A. Waterhouse R. M. Ioannidis P. Kriventseva E. V. Zdobnov E. M. (2015). BUSCO: Assessing Genome Assembly and Annotation Completeness With Single-Copy Orthologs. Bioinformatics 31, 3210–3212. doi: 10.1093/bioinformatics/btv351 26059717

[B71] Smit A. Hubley R. Green P. (2015). RepeatModeler Open-1.0 (2008-2015). Available at: http://www.repeatmasker.org/RepeatModeler/.

[B72] Speth C. Rambach G. Lass-Flörl C. Howell P. L. Sheppard D. C. (2019). Galactosamino-Galactan (GAG) and Its Multiple Roles in Aspergillus Pathogenesis. Virulence 10, 976–983. doi: 10.1080/21505594.2019.1568174 30667338 PMC8647848

[B73] Stamatakis A. (2014). RAxML Version 8: A Tool for Phylogenetic Analysis and Post-Analysis of Large Phylogenies. Bioinformatics 30, 1312–1313. doi: 10.1093/bioinformatics/btu033 24451623 PMC3998144

[B74] Stanke M. Waack S. (2003). Gene Prediction With a Hidden Markov Model and a New Intron Submodel. Bioinformatics 19, ii215–ii225. doi: 10.1093/bioinformatics/btg1080 14534192

[B75] Sun J. Xia Y. Ming D. (2020). Whole-Genome Sequencing and Bioinformatics Analysis of Apiotrichum Mycotoxinivorans: Predicting Putative Zearalenone-Degradation Enzymes. Front. Microbiol. 11, 1866. doi: 10.3389/fmicb.2020.01866 32849454 PMC7416605

[B76] Tang H. (2015). Jcvi: JCVI Utility Libraries. Zenodo doi: 10.5281/zenodo.31631

[B77] Tarailo-Graovac M. Chen N. (2009). Using RepeatMasker to Identify Repetitive Elements in Genomic Sequences. Curr. Protoc. Bioinf. 25, 4.10.1–4.10.14. doi: 10.1002/0471250953.bi0410s25 19274634

[B78] Urban M. Cuzick A. Seager J. Wood V. Rutherford K. Venkatesh S. Y.. (2020). PHI-Base: The Pathogen-Host Interactions Database. Nucleic Acids Res. 48, D613–D620. doi: 10.1093/nar/gkz904 31733065 PMC7145647

[B79] Urban M. Pant R. Raghunath A. Irvine A. G. Pedro H. Hammond-Kosack K. E. (2015). The Pathogen-Host Interactions Database (PHI-Base): Additions and Future Developments. Nucleic Acids Res. 43, D645–D655. doi: 10.1093/nar/gku1165 25414340 PMC4383963

[B80] Van Etten J. Bhattacharya D. (2020). Horizontal Gene Transfer in Eukaryotes: Not If, But How Much? Trends Genet. 36, 915–925. doi: 10.1016/j.tig.2020.08.006 33012528

[B81] Varoquaux N. Liachko I. Ay F. Burton J. N. Shendure J. Dunham M. J.. (2015). Accurate Identification of Centromere Locations in Yeast Genomes Using Hi-C. Nucleic Acids Res. 43, 5331–5339. doi: 10.1093/nar/gkv424 25940625 PMC4477656

[B82] Verma M. Kulshrestha S. Puri A. (2017). Genome Sequencing. Methods Mol. Biol. 1525, 3–33. doi: 10.1007/978-1-4939-6622-6_1 27896715

[B83] Walker B. J. Abeel T. Shea T. Priest M. Abouelliel A. Sakthikumar S.. (2014). Pilon: An Integrated Tool for Comprehensive Microbial Variant Detection and Genome Assembly Improvement. PloS One 9, e112963. doi: 10.1371/journal.pone.0112963 25409509 PMC4237348

[B84] Wang J. Gao Q. Bao J. (2016). Genome Sequence of Trichosporon Cutaneum ACCC 20271: An Oleaginous Yeast With Excellent Lignocellulose Derived Inhibitor Tolerance. J. Biotechnol. 228, 50–51. doi: 10.1016/j.jbiotec.2016.04.043 27130500

[B85] Yu S. Huo K. (2018). Aspergillus Fumigatus Phospholipase D may Enhance Reactive Oxygen Species Production by Accumulation of Histone Deacetylase 6. Biochem. Biophys. Res. Commun. 505, 651–656. doi: 10.1016/j.bbrc.2018.09.157 30286953

[B86] Zhang H. Kang L. Yao H. He Y. Wang X. Xu W.. (2016). *Streptococcus Pneumoniae* Endopeptidase O (PepO) Elicits a Strong Innate Immune Response in Mice *via* TLR2 and TLR4 Signaling Pathways. Front. Cell Infect. Microbiol. 6, 23. doi: 10.3389/fcimb.2016.00023 26973817 PMC4770053

[B87] Zhao S. Guo Y. Sheng Q. Shyr Y. (2014). Heatmap3: An Improved Heatmap Package With More Powerful and Convenient Features. BMC Bioinf. 15, 1–2. doi: 10.1186/1471-2105-15-S10-P16

