# Supplementary material for: Complete Genome Sequencing and Comparative Analysis of the Clinically-Derived Apiotrichum mycotoxinivorans Strain GMU1709
Source: Front Cell Infect Microbiol. 2022 Feb 4;12:834015. doi: 10.3389/fcimb.2022.834015 (PMC8855340; doi:10.3389/fcimb.2022.834015)
Supplement: Supplementary file 1 [file DataSheet_1.docx]

Supplementary Material

# Complete Genome Sequencing and Comparative Analysis of the Clinically-Derived *Apiotrichum mycotoxinivorans* Strain GMU1709

**Liang Peng^1,2,#^, Chen-Fei Liu^1#^, Hong Wu^3,#^, Hai Jin^1^, Xiao-Yan Deng^2^, Li-Ting Zeng^1^, Yi Xiao^1^, Cong Deng^3^, Zhi-Kai Yang^1*^**

^1^The Fifth Affiliated Hospital of Guangzhou Medical University, Guangzhou, China

^2^KingMed School of Laboratory Medicine, Guangzhou Medical University, Guangzhou, China

^3^The Second Affiliated Hospital of Guangzhou Medical University, Guangzhou, China;

^#^These authors contributed equally to this work.

^*^Corresponding author:

Zhi-Kai Yang, Tel./Fax. +86-20-82029637. E-mail: [yangzk@gzhmu.edu.cn](mailto:yangzk@gzhmu.edu.cn)

# Supplementary Figures and Tables

## Supplementary Figures


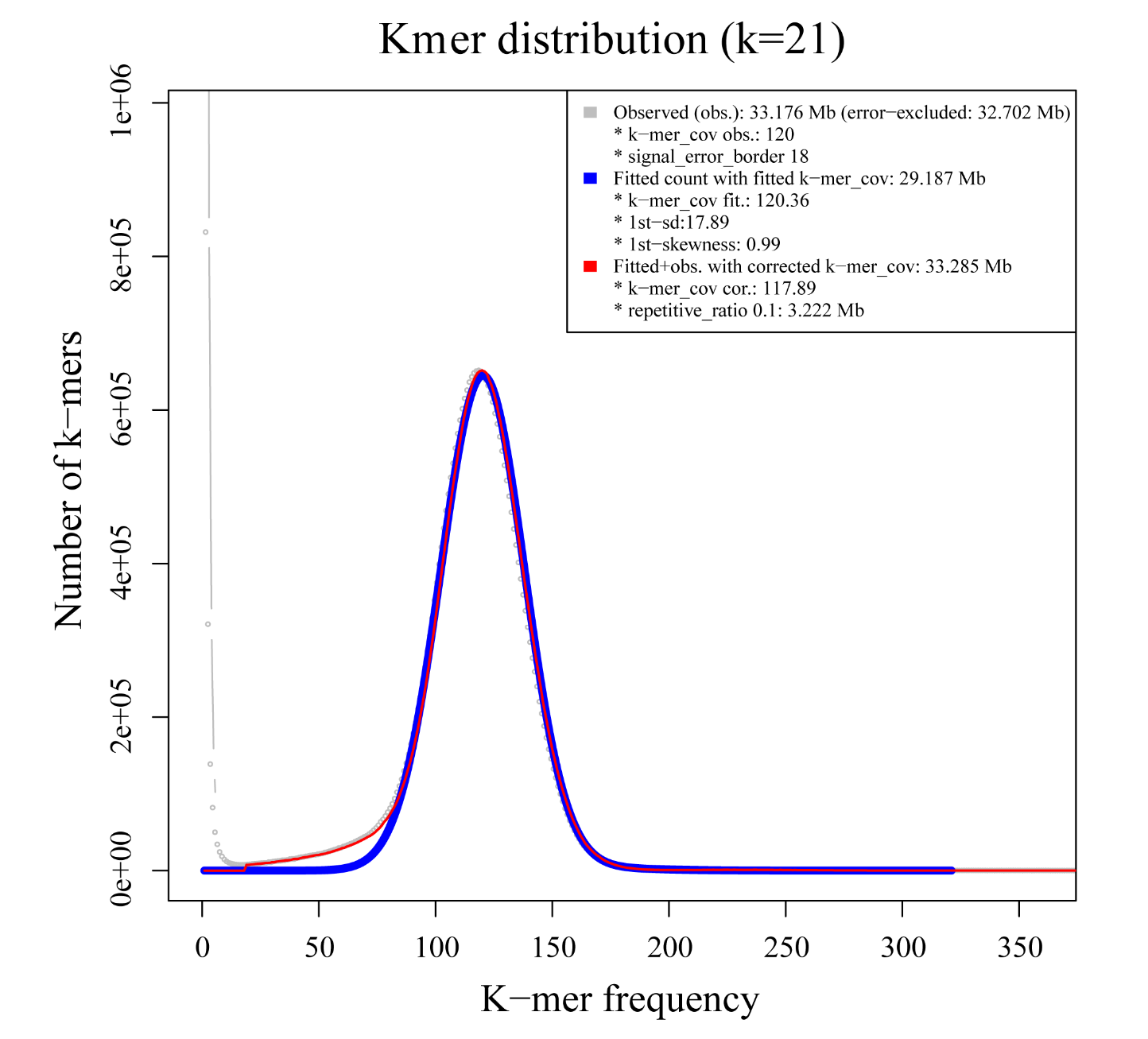


**Figure S1.** Distribution of 21-mer frequency. The number of k-mers (k=21) is plotted against their frequencies. The genome size is estimated to be 33.18 Mb, 29.19 Mb, and 33.29 Mb respectively based on the actual, fitted, and fitted+observed data.


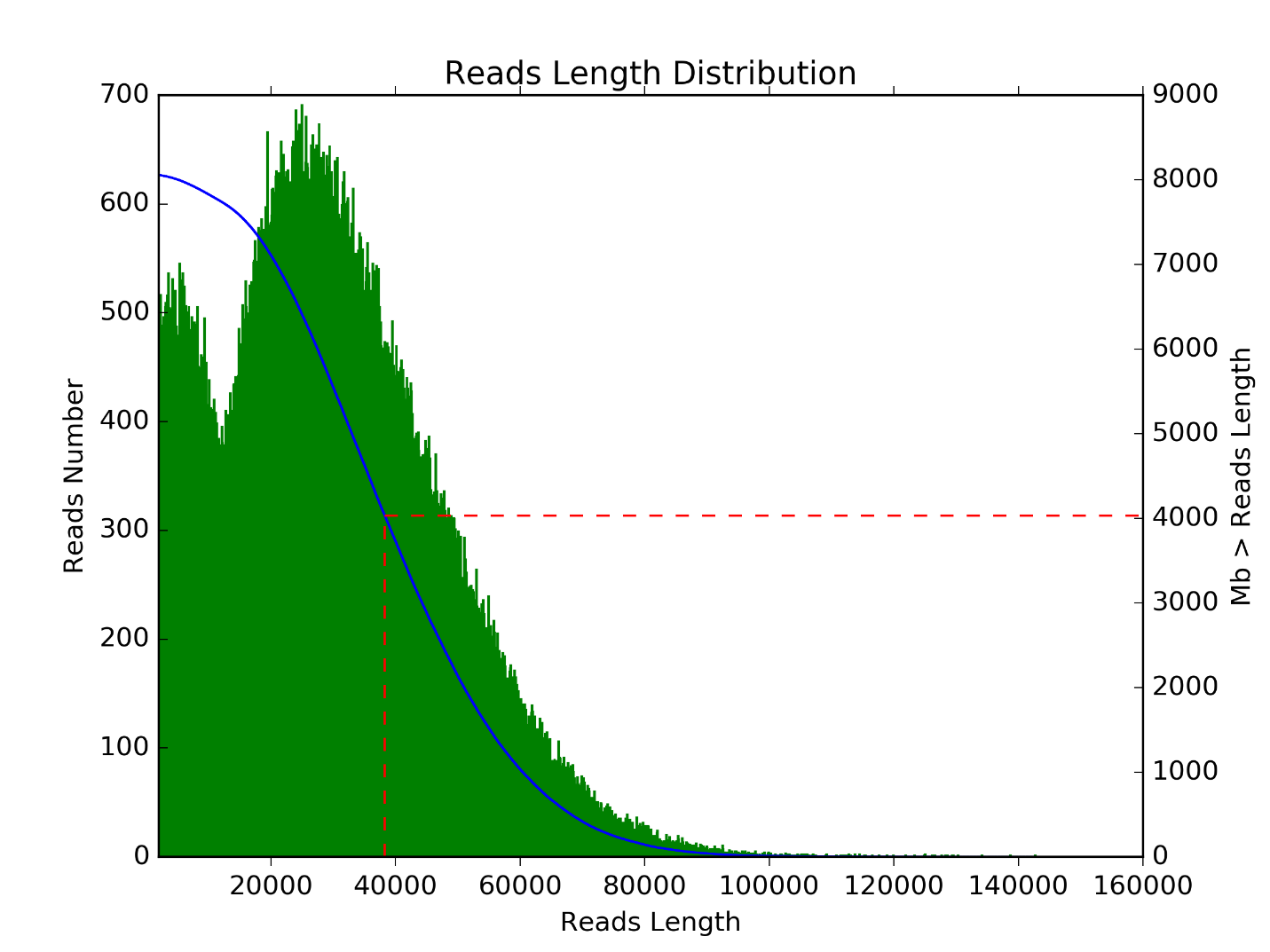


**Figure S2.** Length distribution of Nanopore reads. Right y-axis corresponds to the blue line, and represents the cumulative length of Nanopore reads.


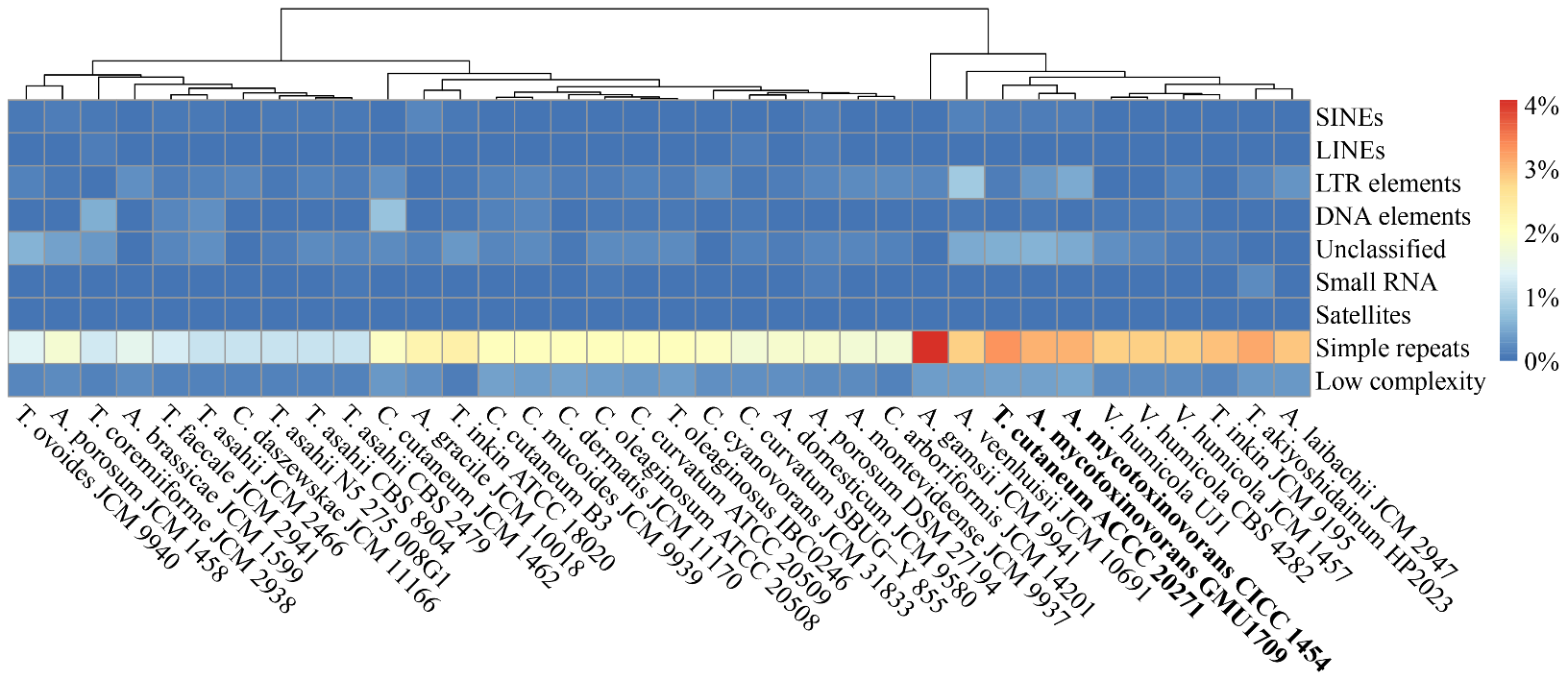


**Figure S3.** Percentage distribution of each type of repeat sequence in 36 strains. SINEs, LINEs, and LTR are the abbreviation of short interspersed nuclear elements, long interspersed nuclear elements, and long terminal repeat retrotransposons, respectively. Unclassified refers to the unclassified interspersed repeats.


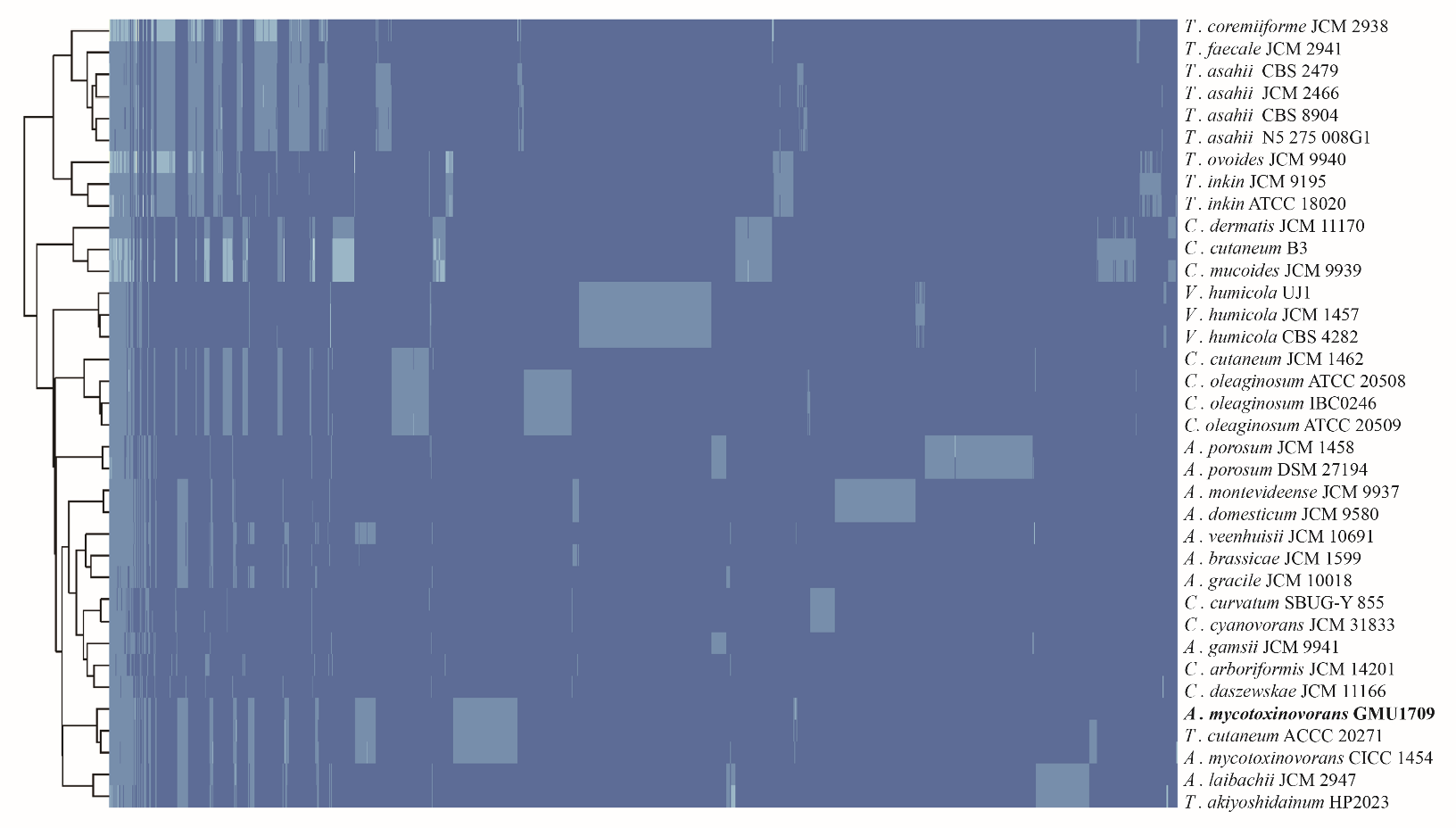


**Figure S4.** Gene presence and absence pattern of 4 genus, 26 species and 36 strains.


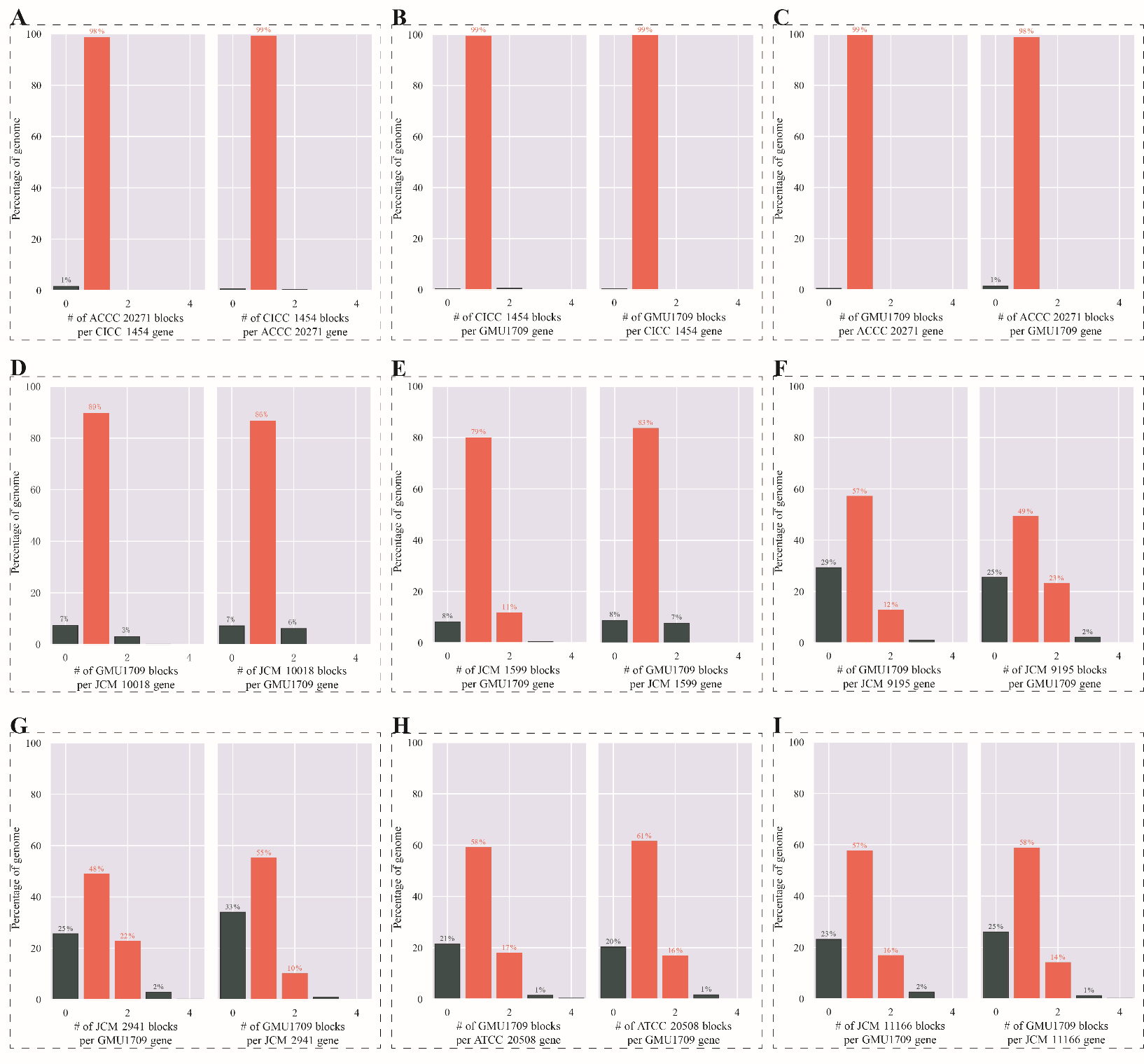


**Figure S5.** Comparison of homology between GMU1709 and strains from the same species, other species, or genus. Intraspecific comparisons of *A. mycotoxinivorans* between ACCC 20271 and CICC 1454 (**A**), CICC 1454 and GMU1709 (**B**), and GMU1709 and ACCC 20271 (**C**). Comparisons of GMU1709 with strains of other species of the same genus, involving in comparisons between GMU1709 and *A. gracile* JCM 10018 (**D**), GMU1709 and *A. brassicae* JCM 1599 (**E**). Comparisons of GMU1709 with strains of *Trichosporon* genus, involving in comparisons between GMU1709 and *T. inkin* JCM 9195 (**F**), GMU1709 and *T. faecale* JCM 2941 (**G**). Comparisons of GMU1709 with strains of *Cutaneotrichosporon* genus, involving in comparisons between GMU1709 and *C. oleaginosum* ATCC 20508 (**H**), GMU1709 and *C. daszewskae* JCM 11166 (**I**).


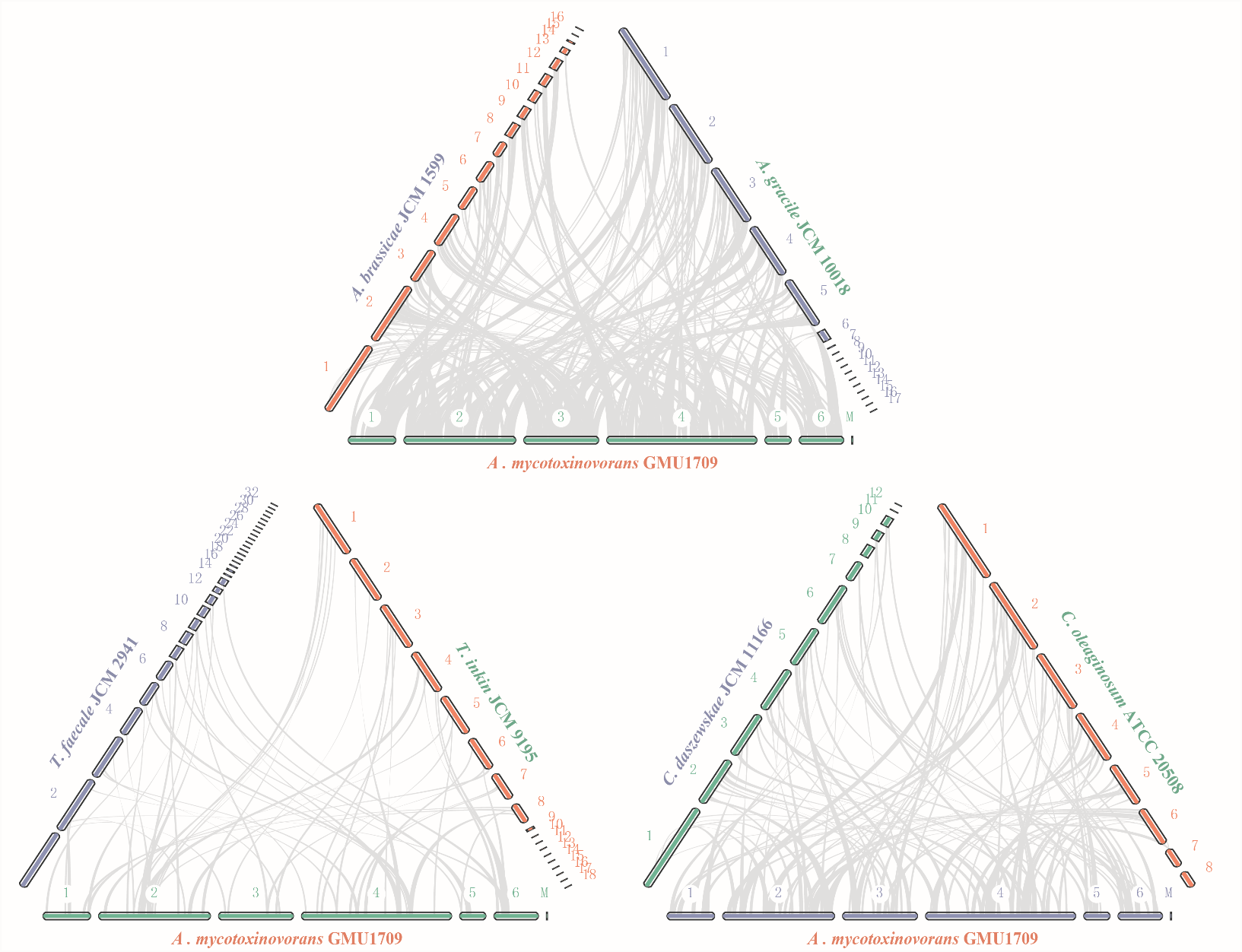


**Figure S6.** Pair-wise synteny blocks between *A. mycotoxinivorans* GMU1709 and strains of other species.


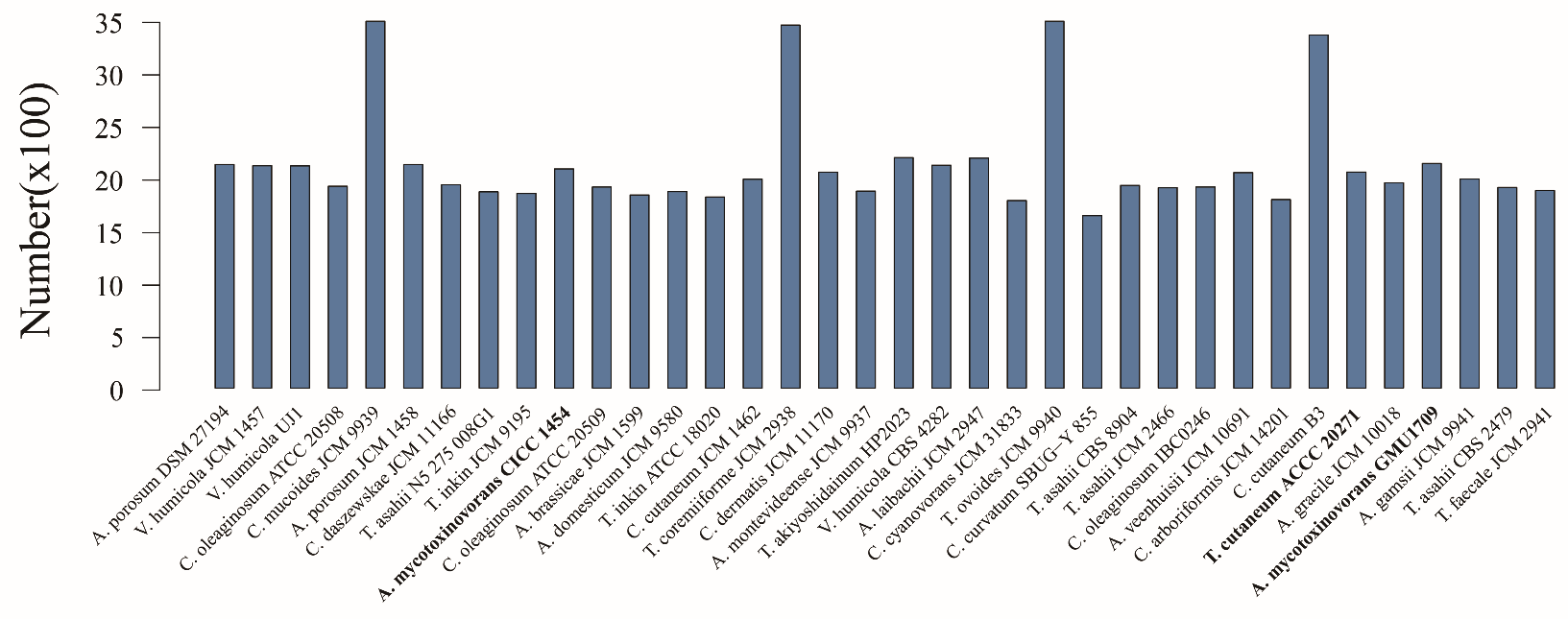


**Figure S7.** Distribution of the number of homologues of PHI gene in 36 *Trichosporonaceae* strains.


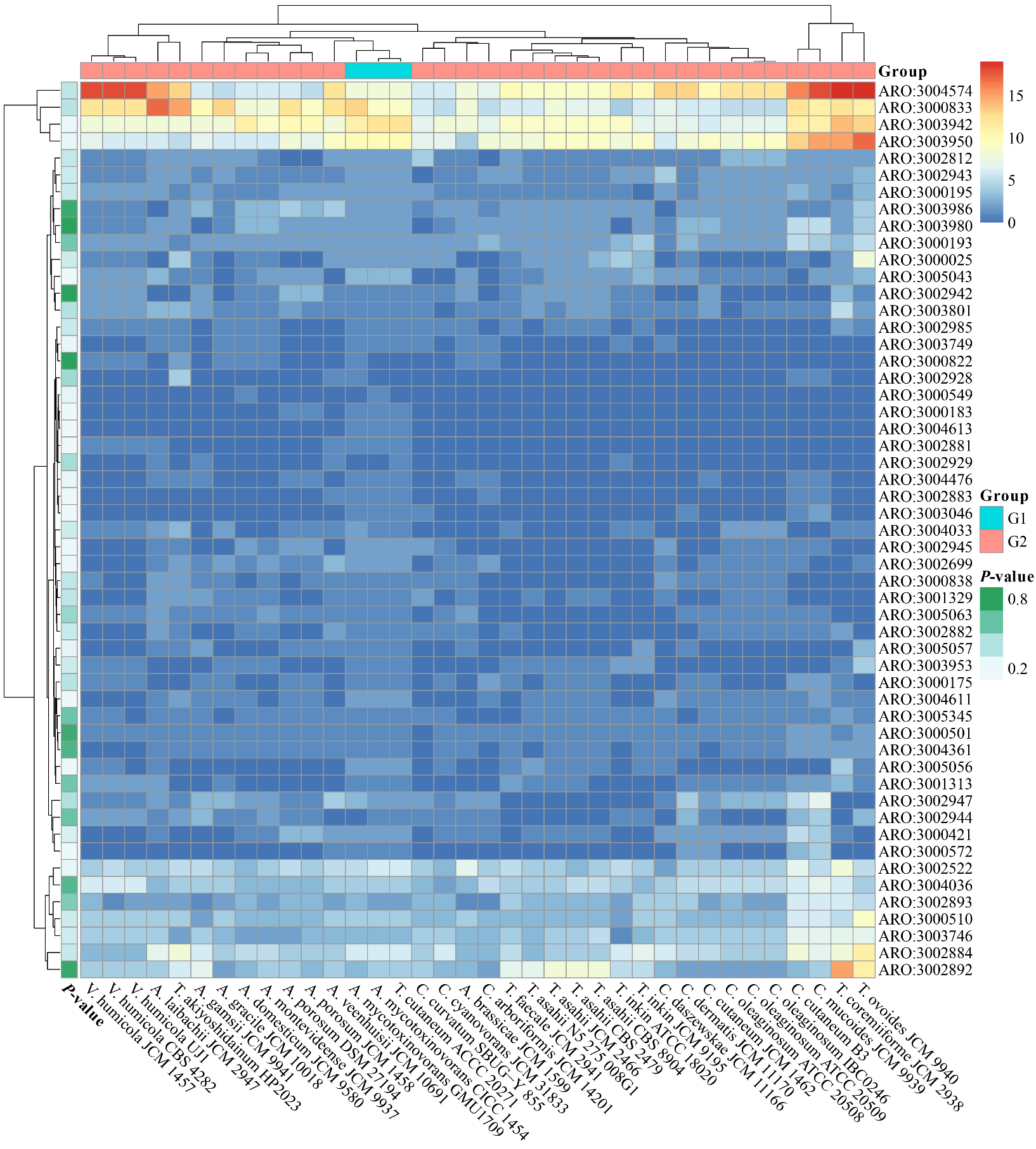


**Figure S8.** Differences (using the Wilcox test) in gene number of antibiotics resistance ontologies between *A. mycotoxinivorans* strains and other *Trichosporonaceae* strains. G1 and G2 groups correspond to the clinical and non-clinical strains, respectively.

**
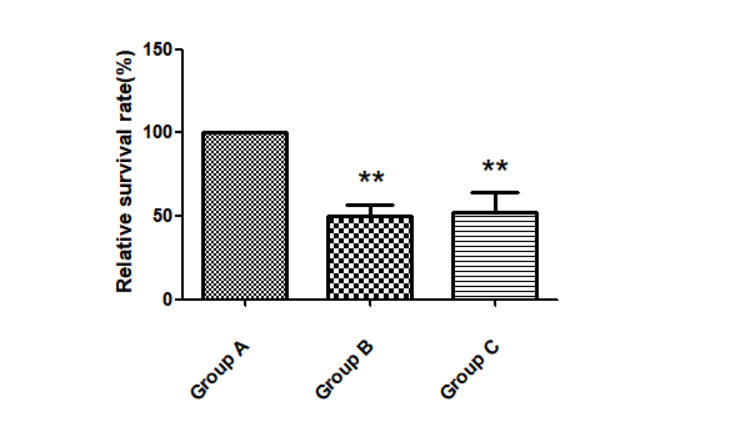
**

**Figure S9.** Resistance of *Escherichia coli* to tetracycline in the presence of *A. mycotoxinivorans* GMU1709. Group A: live GMU1709 mixed with *E. coli*; Group B: inactivated GMU1709 mixed with *E. coli*; Group C: *E. coli* suspended in saline. Error bars indicate standard deviations. ***P*<0.01(compared to Group A).

## Supplementary Tables

**Table S1.** Statistical result of Nanopore reads.

| **Length (bp)** | **Reads Number** | **Total Length (bp)** | **Percent** | **Average Length (bp)** |
| --- | --- | --- | --- | --- |
| 2000~5000 | 14,955 | 52,383,796 | 0.65% | 3502.76 |
| 5000~10000 | 23,545 | 174,599,387 | 2.16% | 7415.56 |
| 10000~20000 | 46,203 | 713,835,198 | 8.85% | 15449.97 |
| 20000~30000 | 62,242 | 1,557,297,538 | 19.32% | 25020.04 |
| 30000~40000 | 52,374 | 1,818,794,650 | 22.57% | 34727.05 |
| 40000~50000 | 35,760 | 1,595,081,418 | 19.79% | 44605.18 |
| 50000~60000 | 20,316 | 1,106,120,201 | 13.72% | 54445.76 |
| 60000~70000 | 9,632 | 619,696,224 | 7.69% | 64337.23 |
| 70000~80000 | 3,708 | 274,794,813 | 3.41% | 74108.63 |
| >=80000 | 1,665 | 145,562,488 | 1.80% | 87424.91 |

**Table S2.** Statistical result of reads mapping back to the *A. mycotoxinivorans* GMU1709 genome.

| **Library size** | **Total reads** | **Mapped (%)** | **Properly mapped (%)** |
| --- | --- | --- | --- |
| 350 bp | 33,134,148 | 94.45 | 92.17 |

**Table S3.** Gene number distribution of different PHI-base accessions in three *A. mycotoxinivoran* strains.

**Table S4.** The PHI-base accessions with significant differences in the number of genes between *A. mycotoxinivoran* strains and other *Trichosporonaceae* strains

**Table S5.** BUSCO quality assessment of the *A. mycotoxinivorans* GMU1709 genome.

| **Complete BUSCOs(C)** | **Complete and single- copy BUSCOs(S)** | **Complete and  duplicated BUSCOs(D)** | **Fragmented BUSCOs(F)** | **Missing BUSCOs(M)** | **Total Lineage BUSCOs** |
| --- | --- | --- | --- | --- | --- |
| 276 (95.17%) | 275 (94.83%) | 1 (0.34%) | 3 (1.03%) | 11 (3.79%) | 290 |
